# Structure and dynamics of the active Gs-coupled human secretin receptor

**DOI:** 10.1038/s41467-020-17791-4

**Published:** 2020-08-18

**Authors:** Maoqing Dong, Giuseppe Deganutti, Sarah J. Piper, Yi-Lynn Liang, Maryam Khoshouei, Matthew J. Belousoff, Kaleeckal G. Harikumar, Christopher A. Reynolds, Alisa Glukhova, Sebastian G. B. Furness, Arthur Christopoulos, Radostin Danev, Denise Wootten, Patrick M. Sexton, Laurence J. Miller

**Affiliations:** 1 Department of Molecular Pharmacology and Experimental Therapeutics, Mayo Clinic, Scottsdale, AZ 85259 USA; 2 School of Biological Sciences, University of Essex, Colchester, CO4 3SQ UK; 3 Drug Discovery Biology Theme, Monash Institute of Pharmaceutical Sciences, Monash University, Parkville, VIC 3052 Australia; 4 Department of Molecular Structural Biology, Max Planck Institute of Biochemistry, 82152 Martinsried, Germany; 5 Graduate School of Medicine, University of Tokyo, N415, 7-3-1 Hongo, Bunkyo-ku 113-0033 Tokyo, Japan; 6 Present Address: Centre for Sport, Exercise and Life Sciences, Faculty of Health and Life Sciences, Alison Gingell Building, Coventry University, CV1 2DS Coventry, UK; 7 Present Address: Novartis Institutes for Biomedical Research, Novartis Pharma AG, 4002 Basel, Switzerland

**Keywords:** Cryoelectron microscopy, Structural biology, Gastroenterology

## Abstract

The class B secretin GPCR (SecR) has broad physiological effects, with target potential for treatment of metabolic and cardiovascular disease. Molecular understanding of SecR binding and activation is important for its therapeutic exploitation. We combined cryo-electron microscopy, molecular dynamics, and biochemical cross-linking to determine a 2.3 Å structure, and interrogate dynamics, of secretin bound to the SecR:Gs complex. SecR exhibited a unique organization of its extracellular domain (ECD) relative to its 7-transmembrane (TM) core, forming more extended interactions than other family members. Numerous polar interactions formed between secretin and the receptor extracellular loops (ECLs) and TM helices. Cysteine-cross-linking, cryo-electron microscopy multivariate analysis and molecular dynamics simulations revealed that interactions between peptide and receptor were dynamic, and suggested a model for initial peptide engagement where early interactions between the far N-terminus of the peptide and SecR ECL2 likely occur following initial binding of the peptide C-terminus to the ECD.

## Abstract (web summary)

The class B secretin GPCR (SecR) has broad physiological effects, with target potential for treatment of metabolic and cardiovascular disease. Here, authors present a cryo-EM structure and biochemical studies of secretin binding to the SecR:Gs complex which show that interactions between peptide and receptor were dynamic.

## Introduction

G protein-coupled receptors (GPCRs) are the largest family of cell surface receptors and pre-eminent drug targets. There are four major subclasses of GPCRs: A, B, C, and F. Peptide hormone class B GPCRs are a subfamily of GPCRs that are particularly important in physiology and disease, since their endogenous ligands play major roles in homeostatic control of bone and energy metabolism, cardiovascular, and immune responses^1^. Consequently, these receptors are important targets for treatment of disorders of these functions. Class B GPCRs encompass targets for approved drugs that treat diabetes, obesity, osteoporosis, hypercalcemia, and Paget’s disease, all of which are major global health burdens^1^. However, targeting these receptors for therapeutic benefit is suboptimal^1^. Class B receptors are also pleiotropically coupled and we currently lack a complete understanding of the breadth of signaling that is required for specific clinical efficacy and how this can be optimally achieved.

The class B secretin GPCR (SecR) is renowned for its physiological role as a regulator of pancreatic and biliary ductular epithelial secretion^2^. Subsequent studies, including phenotypic analysis of mice genetically engineered to have no secretin peptide (Sec^−/−^)^3^ or receptor (SecR^−/−^)^4^, have revealed a much broader profile of action and the potential for secretin receptor agonists to fill key unmet clinical need across a range of diseases including obesity and diabetes, as well as heart failure^5^, among the most prevalent, costly, and debilitating public health problems. For example, secretin produces satiety to reduce body weight^6^, has direct thermogenic effects on adipocytes^7^ and elicits a glucose-sensitive incretin effect to help normalize glucose^8^. Further, secretin increases cardiac output and stroke volume and reduces systemic vascular resistance, while increasing coronary, renal, mesenteric, and carotid flow, providing benefits for heart failure^5^. Understanding the molecular basis for secretin receptor binding and activation is therefore important for therapeutic exploitation of this receptor.

In this study, we have combined single-particle cryo-electron microscopy, molecular dynamics (MD), and biochemical cross-linking to determine the structure and dynamics of secretin bound to the human SecR:Gs protein complex. While there are parallels to other active class B peptide hormone receptor structures, the SecR demonstrates a unique organization of the receptor extracellular domain (ECD) to the 7-transmembrane (TM) domain core, forming more extended interactions than other class B GPCRs. Secretin formed numerous polar interactions between the N-terminal half of the peptide and receptor extracellular loops (ECLs) and TM helices, with the importance of these interactions supported by mutagenesis data. Cysteine-cross-linking analysis and MD simulations revealed that the interactions between SecR and secretin were dynamic, and suggested a model for initial peptide engagement where early interactions between the far N-terminus of the peptide and the SecR ECL2 likely occur following initial binding of the peptide C-terminus to the receptor ECD.

## Results

### Cryo-EM determination of the secretin:SecR:Gs complex

**Fig. 1:**
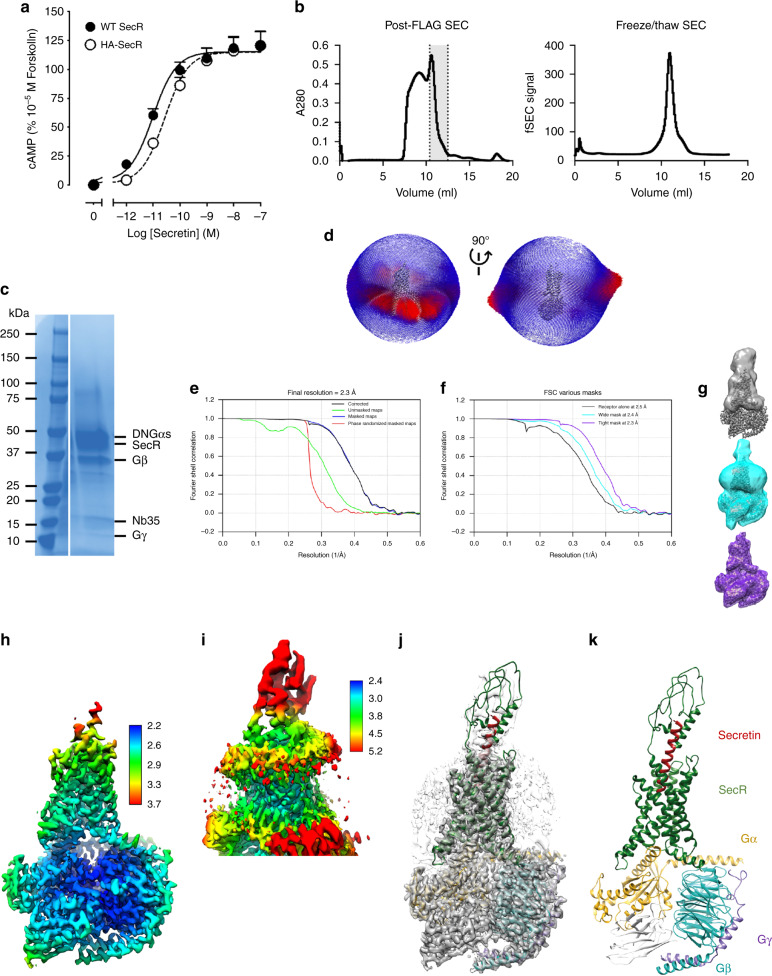
Cryo-EM structure of the secretin:SecR:DNGα_s_v2:Nb35 complex. **a** Pharmacology of the expression construct for HA-SecR versus WT-SecR (WT-SecR, pEC50 11.0 ± 0.1; HA-SecR, pEC50 10.6 ± 0.2; *n* = 4). **b** SEC trace of post FLAG-affinity column elution (left panel), the complex peak (gray, dotted lines) was isolated and used for cryo-EM imaging, with the right panel illustrating stability of the complex following one cycle of freeze-thawing. **c** Coomassie stain of the purified complex separated by PAGE (right panel). **d** 3-D histogram representation of the Euler angle distribution of all the particles used in the reconstruction overlaid on the density map drawn on the same coordinate axis (shown from the front and 90° rotated). **e** Gold standard Fourier shell correlation (FSC) curves for the global, postprocessed map using a tight mask showing the overall nominal resolution of 2.3 Å. **f** Corrected FSC curves of maps using different masking: consensus refinement, postprocessed using a tight mask (excluding ECD and micelle, 2.3 Å, in purple), consensus refinement, postprocessed using a wide mask (2.4 Å, in cyan), and local refinement with using a receptor-only mask (2.5 Å, in gray). **g** Masks used for calculating FSC curves in **f**, displayed onto the global refinement map (colored according to **f**). **h** Local resolution-filtered EM map displaying local resolution (in Å) colored from highest resolution (blue) to lowest resolution (red) of the global map. **i** Local resolution-filtered EM map displaying local resolution (in Å) of the receptor-only refinement. **j** Global map (dark gray) overlaid with receptor-only map (gray transparent) containing the backbone model of the complex in ribbon format; SecR (dark green), secretin (dark red), G protein α-subunit (gold), β-subunit (cyan), γ-subunit (dark purple), and Nb35 (white). **k** Ribbon representation of the secretin:SecR:DNGα_s_v2:Nb35 complex colored according to **j**.

The human SecR was modified to replace the native signal sequence with that of hemagglutinin (HA), followed by a Flag epitope, and inclusion of a C-terminal His tag, both flanked by 3C cleavage sites, as previously described for other class B GPCRs^9^. The expression construct maintained an equivalent ability to wild-type receptor to signal to Gs-mediated cAMP production (Fig. 1a). Complexes of the receptor with dominant negative Gα_S_:Gβ_1_γ_2_^9,10^ were formed by the addition of 1 μM secretin. Two distinct datasets were collected >12 months apart, however, the biochemistry for formation of the two complexes was equivalent, except that the Gαs protein contained an additional A366S mutation (DNGα_s_v2) for the latter complex^10,11^. The complexes exhibited a monodisperse peak on size-exclusion chromatography (SEC) (Fig. 1b, Supplementary Fig. 1a, right panels), following purification by anti-Flag antibody chromatography and an initial SEC separation (Fig. 1b, Supplementary Fig. 1a, left panels), containing each of the component proteins (Fig. 1c, Supplementary Fig. 1b). The complexes were imaged by single-particle cryo-EM. The first data set yielded a map of FSC 0.143, 4.3 Å global resolution, and this was used to build the initial model used for MD simulations (Supplementary Fig 1d–f). A second data set was collected using improved vitrification and imaging protocols established subsequently in the Danev laboratory^11^. Only the high-resolution structure is described below; however, the original model derived from the lower resolution map exhibited high overall concordance with the model constructed into the high-resolution map (Supplementary Fig. 1g–i).

Although there was preferred orientation of the particles (Fig. 1d), these data were processed to yield final maps with global resolutions, by gold standard FSC 0.143, of 2.3–2.5 Å (Fig. 1e, f). The highest local resolution was present for the receptor transmembrane domain, G protein, and for the peptide N-terminus that binds deep into the receptor core, and this was reflected in the 2.3 Å (tight mask excluding micelle and Gs α-helical domain (AHD)) and 2.4 Å (wide mask) maps (Fig. 1h, i). An atomic model of the complex was built into the electron density map using MD-guided fitting and manually inspected and adjusted using geometric constraints (Supplementary Fig. 2). Both the high-resolution map with the tight mask (Fig. 1g, h) and the receptor-focused map (Fig. 1g, i) were used in modeling, since the latter had better resolution for the loops and peptide C-terminus (Supplementary Fig. 2). Overall, the maps allowed accurate placement of side-chain rotamers for most of the receptor transmembrane domain, all ECLs, ICL1 and ICL2, and G protein, as well as the N-terminus of the peptide (Supplementary Fig. 2); however, the ECD resolution did not allow unambiguous placement of side chains, and only the backbone was modeled between R30^ECD^ (the first modeled amino acid) and S130^ECD^. Similarly, residues 254–263 of Gα_s_ were only modeled as a backbone trace, while the AHD of Gα_s_ (62–204) was omitted from the model. We also performed extensive MD simulations (3 × ~1 μs) to derive further insight into binding dynamics of the secretin peptide to the receptor and engagement of the receptor with the Gs protein.

### General features of the secretin:SecR:Gs complex

**Fig. 2:**
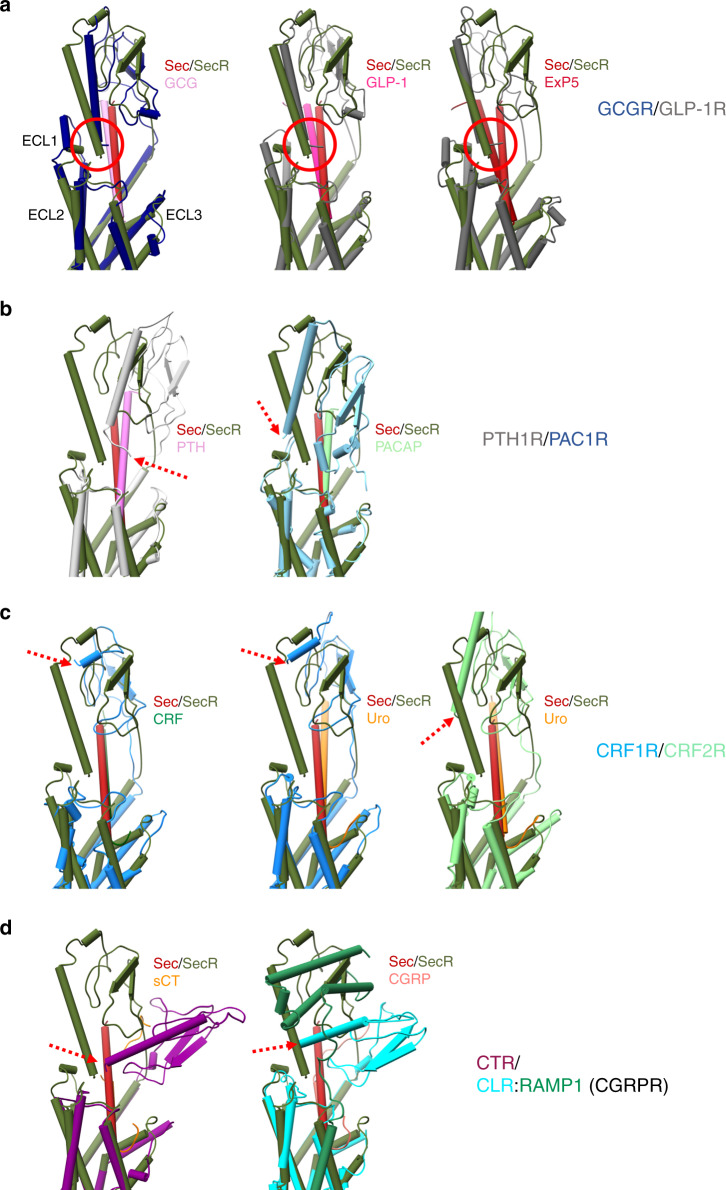
Comparison of SecR and other class B GPCR Gs-coupled active structures. Overlay of secretin (dark red) and SecR (dark green) and different class B GPCR subfamilies emphasizing the position of the receptor ECD and location of the far N-terminus of the receptor (highlighted by red circle (**a**) or red arrows (**b**–**d**). **a** Glucagon (GCG, pink; GCGR dark blue) and GLP-1 receptors (GLP-1, dark pink; ExP5, red; GLP-1R, dark gray). **b** PTH1 (PTH, pink; PTH1R, light gray) and PAC1 receptors (PACAP38, pale green; PAC1R, light blue). **c** CRF1 (CRF, dark green; Urocortin (Uro), orange; CRF1R, blue) and CRF2 receptors (Uro, orange; CRF2R, green). **d** Calcitonin (sCT, orange; CTR, purple) and CGRP (CGRP, coral; CLR, cyan; RAMP1, green) receptors. Helical secondary structure is shown as cylinders; beta sheets are displayed as flattened arrows.

The active SecR complex exhibits the key features of active class B GPCRs with outward movement of the tops of TM6/TM7/ECL3, lateral movement of TM1 and reordering of ECL2 into a common fold (Fig. 1j, k, Supplementary Fig. 3)^12^, paralleled by a large outward movement of TM6 at the base of the receptor to accommodate G protein binding (Supplementary Fig. 3c). The secretin peptide forms an extended α-helix that exits the receptor almost perpendicular to the membrane, extending out of the transmembrane domain core (Figs. 1j, k and 2, Supplementary Fig. 4). The N-terminus terminates above the conserved class B receptor central polar network, similar to the related GCG family of peptides at their cognate receptors^9,12,13^ and forms extensive interactions with TM1, TM2, TM5, TM6, and TM7 (Supplementary Fig. 3D, Supplementary Tables 1–4).

### SecR ECD

The ECD orientation of class B GPCRs is one of the most variable features observed in active structures^12^, and we have speculated that this may be important in the activity of the individual receptors. The N-terminal helix of the ECD in the SecR extends deep towards the ECL regions of the receptor core where it makes interactions with ECL1, ECL2, and the secretin peptide that likely stabilize the overall dynamics of the ECD (Figs. 1k and 2, Supplementary Fig. 5b–d). Fine angular sampling using the receptor-focused mask identified two distinct classes with altered positioning of the ECD, and this likely contributes to the lower overall resolution in the consensus maps (Supplementary Fig. 5). Dynamic motions of the receptor are discussed further below. The most closely related orientation of the ECD is seen with the GCG receptor family that are the most evolutionarily conserved with SecR, and the far N-terminal helix of these receptors also extends to the top of the ECLs, though not to the extent of the SecR (Fig. 2a). While the PAC1 and PTH1 receptor ECDs exhibit partial overlap in orientation to the SecR, the far N-terminus is oriented further away from the receptor core (Fig. 2b). Interestingly, the location of the ECD for the CRF receptor family overlaps closely with that of the SecR, but they do not have an equivalent N-terminal α-helix and thus lack equivalent interactions with the receptor core (Fig. 2c), and this likely accounts for the lower relative resolution of the ECD in structures of these receptors^12,14^. The calcitonin family receptors have a markedly distinct ECD orientation from other class B GPCRs that is enabled by an unstructured peptide C-terminus (Fig. 2d)^15^. These receptors form prominent interactions with the receptor activity-modifying protein (RAMP) family to yield unique receptor phenotypes for binding of CGRP, adrenomedullin (AM), amylin, and related peptides^16^. Interestingly, the location of the RAMP ECD overlaps the position of the SecR ECD (Fig. 2d, Supplementary Fig. 4). Although the SecR can also interact with RAMP3^17^, the structural data indicates that it is unlikely to engage via an equivalent (TM3, TM4, TM5, and ECD) interface to that of the CT family receptors^15^. This is consistent with our previous work illustrating that it is the transmembrane domain, and not ECD, which is most critical for RAMP3 and SecR dimerization, and that TM6 and TM7 of SecR may form the site of interaction with the RAMP3 TM domain^17^. As all class B GPCR peptides, except those of the CT receptor subgroup, have C-terminal extended α-helices, and thus a principally vertical ECD orientation, similar to SecR (Fig. 2, Supplementary Fig. 4), it is likely that the RAMP engagement that is seen with most other class B GPCRs would follow an interaction model similar to SecR.

### Secretin binding site

**Fig. 3:**
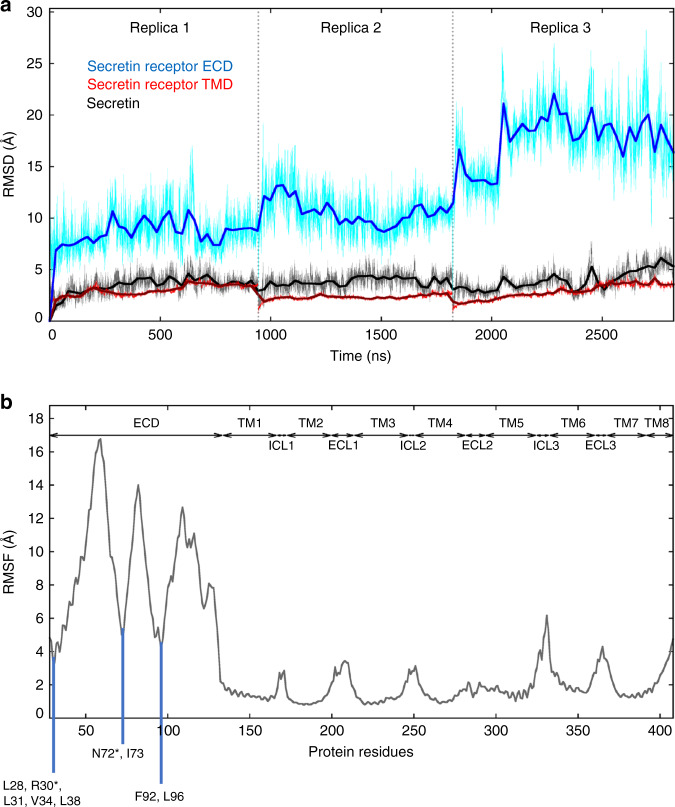
The SecR TM domain and the secretin were stable during MD simulations. **a** The RMSD (starting structure set as reference) of alpha carbon atoms of the secretin, the SecR TMD (residues from K134^1.31^ to L391^7.60^), and the SecR ECD (residues from L28^ECD^ to E133^ECD^) was plotted over the total simulation time (single MD replicas are separated by the dotted lines). The ECD high degree of flexibility did not involve the peptide, and the SecR TMD backbone showed low mobility over the simulations (RMSD usually lower than 3.0 Å). **b** SecR alpha carbons RMSF values during MD simulations. TM helices, intracellular loops (ICLs), and extracellular loops (ECLs) positions are indicated at the top. Areas of lower RMSF in the ECD correspond to regions of stable interaction with secretin peptide during MD simulations with specific interacting residues highlighted. Those with asterisks (*) are predicted to form stable H-bonds (see Supplementary Tables 2, 3).

Dynamics of the SecR complex was examined by 3D multivariate analysis of the cryo-EM data and in the long time-scale MD simulations. Separation of the conformational variance in the cryo-EM data into the three main principal components revealed that receptor exhibits twisting and rocking motions that are apparent relative to the G protein. These motions are similar to but less dynamic than those previously observed for the TM core of AM receptors^11^. Across each of the principal components, the receptor ECD exhibited higher relative motion than the rest of the receptor but was nonetheless restricted by interactions with the peptide and receptor core, where the far N-terminus made dynamic interactions with the ECLs; however, no substantial translational motions were evident (Video 1). This contrasted to the much broader motions that were previously observed for the ECD of AM receptors using equivalent analyses^11^. Importantly, the cryo-EM conformational variance data were consistent with the MD simulations where, overall, the receptor TM domain core and the secretin peptide exhibited limited dynamic motion of the peptide backbone, whereas the ECD exhibited higher mobility that was independent of motions of the rest of the receptor and peptide (Fig. 3a, Video 2). Within the ECD, those regions that maintained stable contacts with the secretin peptide had substantially less motion than the rest of the ECD that lacked these constraints (Supplementary Tables 2–4, Fig. 3b, Video 2).

**Fig. 4:**
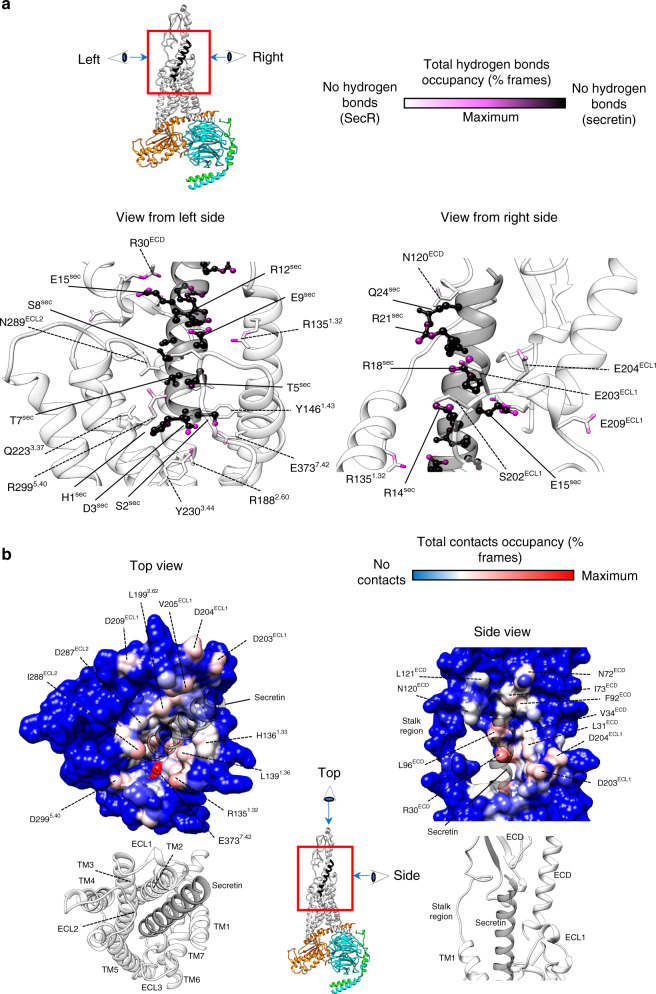
Interactions between SecR and secretin during MD simulations. **a** Hydrogen bonds between SecR and secretin. The total occupancy (% frames) of each atom is plotted onto the equilibrated complex according to a color scale, with SecR atoms never involved in white, secretin atoms never involved in black and atoms highly involved in magenta. The peptide (ball and stick residues indicated with solid lines) is depicted as black, partially transparent ribbon, while the receptor (stick residues indicated with dashed lines) is shown as white, partially transparent ribbon. The central image specifies the relative perspectives from the right and left sides. Right side view) Hydrogen bonds between the N-terminus of secreting and the TM bundle of the SecR; the main interactions involved S2^sec^—E373^7.42^, D3^sec^—R188^2.60^, and E9^sec^—R135^1.32^. Left side view) Hydrogen bonds between the C-terminus of secretin and the ECL1 of the SecR; main intermolecular interactions involved R14^sec^, R18^sec^, R21^sec^ on the peptide, and D203^ECL1^, D204 ^ECL1^, and D209 ^ECL1^ on the receptor. **b** Contacts between SecR and secretin. The total occupancy (% frames) of each atom is plotted onto the surface of the equilibrated SecR according to a color scale, with SecR atoms never involved in blue, and atoms highly involved in red. The peptide is shown as transparent gray ribbon. The central image specifies the relative perspectives from the top and side. Top view) Contacts between secretin and the TM bundle of the SecR (residues from L28^ECD^ to E133^ECD^ have been removed for clarity); the bottom figure shows the ribbon representation as reference. Main interactions involved TM1 (R135^1.32^, H136^1.^^33^, and L139^1^^.36^), TM2 (L199^2.71^), ECL2 (D287^ECL2^ and I288^ECL2^), TM5 (D299^5.40^), and TM7 (E373^7.42^). Side view) Contacts between secretin C-terminal and SecR; the bottom figure shows the ribbon representation as reference. Main interactions involved ECL1 (D203^ECL1^, D204^ECL1^, and D209 ^ECL1^), and ECD (R30^ECD^, L31 ^ECD^, V34 ^ECD^, F92^ECD^, I73^ECD^, and L96^ECD^).

Atomic modeling into the static consensus high-resolution maps revealed specific details on the interactions between secretin and SecR and these are reported in (Supplementary Table 1, Supplementary Fig 6a, b). To better understand these interactions, we interrogated their stability in a simulated POPC lipid environment over microseconds of MD. The secretin peptide forms an amphipathic α-helix and contains many polar and charged amino acids, and perhaps not surprisingly forms extensive stable and transient H-bond interactions over the course of the microsecond simulations, particularly with the receptor core and ECLs. The first three amino acids of secretin are among the most critical for receptor activation^18,19^, and each of these residues forms critical H-bond interactions with the receptor (Supplementary Table 1, Supplementary Fig. 6a, b). In the simulations, His^1P^ and Ser^2P^ (peptide residues are recorded using the three-letter amino acid code, with sequence numbers superscripted) are predicted to form stable backbone and/or side-chain interactions with E373^7.42^ (superscript numbers refer to the Wootten et al. class B numbering system^20^) deep within the receptor core, with His^1P^ forming additional transient interactions with R299^5.40^, Y230^3.44^, Q223^3.37^, and potential weak interactions with the backbone of ECL3 residues E363 and M366 (Fig. 4a, Supplementary Tables 2, 3). Asp^3P^ is predicted to form relatively stable interactions with R188^2.60^ of the central polar network, as well as with R299^5.40^ and Y146^1.43^ (Fig. 4a, Supplementary Table 2); the latter are distinct from the interactions observed in the static consensus structure and highlight the likely importance of conformational dynamics in the action of peptide agonists. The identified class B receptor residues form conserved interactions with most cognate class B peptides^12^, and prior mutagenesis has also demonstrated importance of at least Y146^1.43^, R188^2.60^, and Q223^3.37^ for peptide function in the SecR^21–23^. Gly^4P^, Thr^5P^, Thr^7P^, and Ser^8P^ are predicted to form potential weak H-bonds with residues within ECL2 of the SecR, with the most persistent interactions occurring between Ser^8P^ and N289^ECL2^ (Fig. 4a, Supplementary Tables 2, 3). Glu^9P^ is predicted to form a stable interaction with R135^1.32^, while the cluster of basic residues, Arg^14P^, Arg^18P^, and Arg^21P^ that reside on one face of the secretin peptide form extensive electrostatic and H-bond interactions with a cluster of acidic Asp residues within or adjacent to ECL1, D196^2.68^, D203^ECL1^, D204^ECL1^, and D209^ECL1^ (Fig. 4a, Supplementary Tables 2, 3). In contrast to the extensive polar interactions with the receptor core, only relatively few H-bond interactions were predicted between the secretin peptide and SecR ECD; these were between Glu^15P^ and R30^ECD^, Gln^24P^ and N120^ECD^, and the backbone of Leu^26P^, and to a lesser extent Val^27P^ and N72^ECD^ (Fig. 4a, Supplementary Tables 2, 3), although these interactions were predicted to limit mobility within the ECD as noted above (Fig. 3b).

In addition to the polar interactions described above, there were extensive predicted hydrophobic interactions between the peptide and receptor, particularly from the lipophilic face of the peptide (Phe^6P^, Leu^10P^, and Leu^13P^) that made broad interactions with aliphatic side chains of residues in TM7 and TM1 (L374^7.43^, H136^1.33^, L139^1.36^, L142^1.39^, and K143^1.40^) (Fig. 4b, Supplementary Table 4). Within the ECD there was a hydrophobic surface comprised of the aliphatic side chains of L28^ECD^, R30^ECD^, L31^ECD^, V34^ECD^, and L38^ECD^ of the N-terminal α-helix, and loop residues N72^ECD^, I73^ECD^, F92^ECD^, and L96^ECD^ that provide the principal ECD binding groove for the peptide and interacted with Leu^19P^, Gln^20P^, Leu^22P^, Leu^23P^, Leu^26P^, and Val^27P^ (Fig. 4b, Supplementary Table 4). Moreover, the simulations predicted that Arg^18P^ and Leu^22P^ could form interactions with both ECL1 and the ECD (Supplementary Table 4) potentially contributing to stability of the location and dynamics of ECD-core interactions.

We have previously performed cysteine scanning mutagenesis of ECLs 1, 2, and 3 and analyzed these for effect on cell surface receptor expression, secretin affinity and secretin efficacy in cAMP production^23,24^ (Supplementary Table 5). In the current study this analysis was extended to include residues at the top of TM1 (Supplementary Table 6). Strikingly, mutation of residues in ECL1 and its proximal extension to TM2 had the greatest impact on both secretin affinity and potency (Supplementary Fig 7a, b), consistent with the key role of this receptor region in interactions with both the secretin peptide and the far N-terminus of the ECD. Similarly, receptor residues in ECL2 and ECL3 that formed side-chain interactions with the peptide impacted on either peptide affinity or potency (Supplementary Fig. 7a, b). Interestingly, although mutations to residues in TM1 had relatively limited impact on secretin potency (Supplementary Fig. 7a), there was increased apparent affinity of secretin with mutation of residues towards the apex of TM1 but which were mostly oriented away from the peptide interaction interface (Supplementary Fig. 7b). It is possible that substitution with the small amino acid cysteine may have increased flexibility of this region to allow more favorable peptide interactions.

### Cysteine cross-linking of Cys-substituted secretin peptides

**Fig. 5:**
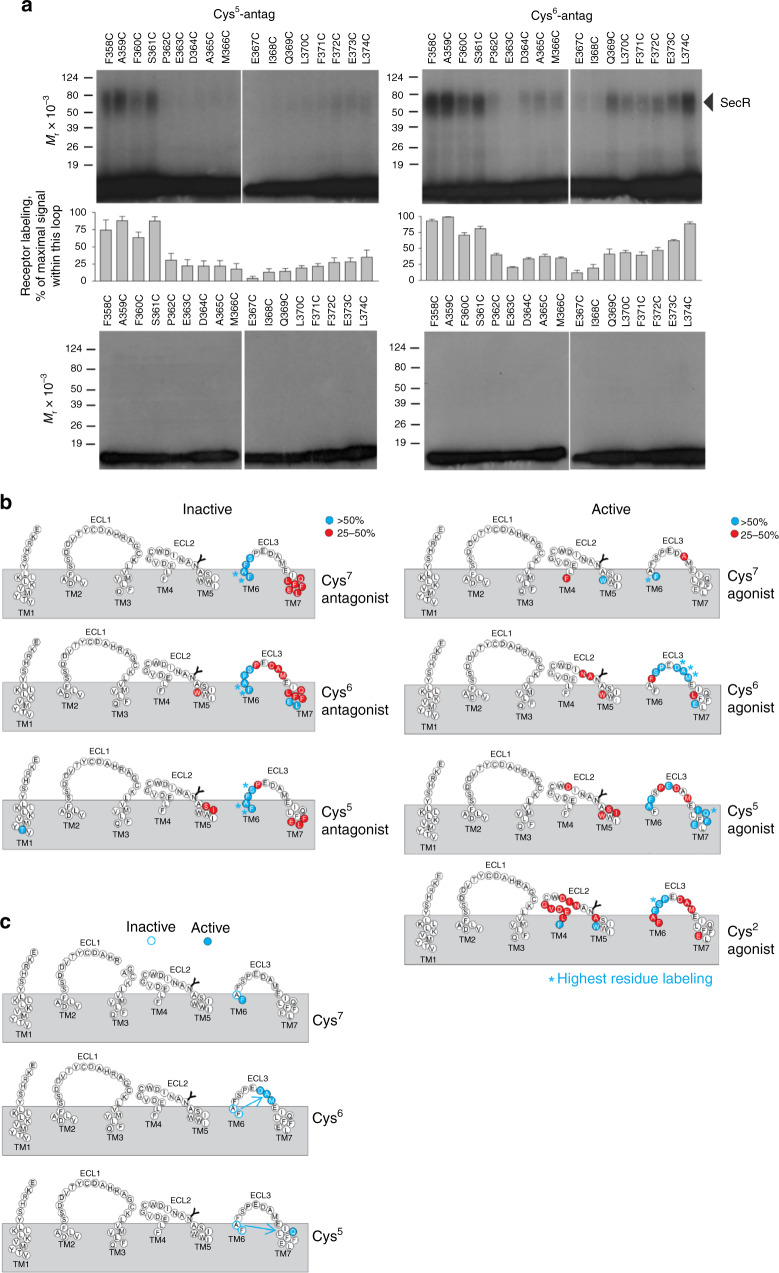
Overview of cysteine-cross-linking by analogous cysteine-substituted agonist and antagonist secretin peptides. **a** Cysteine trapping of secretin receptor ECL3 cysteine replacement mutants with ^125^I-labeled Cys^5^- or Cys^6^-containing secretin antagonist analogs. Shown are typical autoradiographs of 10% SDS–PAGE gels used to separate the products of cysteine trapping of the indicated ECL3 SecR cysteine replacement mutants transiently expressed in COS-1 cells for each of the noted cysteine-containing secretin antagonist probes, under nonreducing (top panel) and reducing (bottom panel) conditions. Autoradiographs are representative of a minimum of three independent experiments. Densitometric analysis of data from three similar experiments for each probe is shown (middle panel), with intensities representing the percentages of the signal for the maximal labeling of a residue within ECL3 by that probe. **b** Illustration of the difference of labeling within the extracellular loops of the cysteine-substituted secretin receptor mutants by cysteine-containing antagonist (left hand panels) and agonist (right hand panels) probes. Blue colored residues are those with most efficient labeling (>50% of the highest efficiency label), with the highest labeled residue denoted with a blue asterisk. Red colored residues denote those that cross-linked with intermediate efficiency (25–50% of the highest efficiency label). **c** Schematic illustration of the major shifts in residue labeling between equivalent agonist and antagonist probes.

**Fig. 6:**
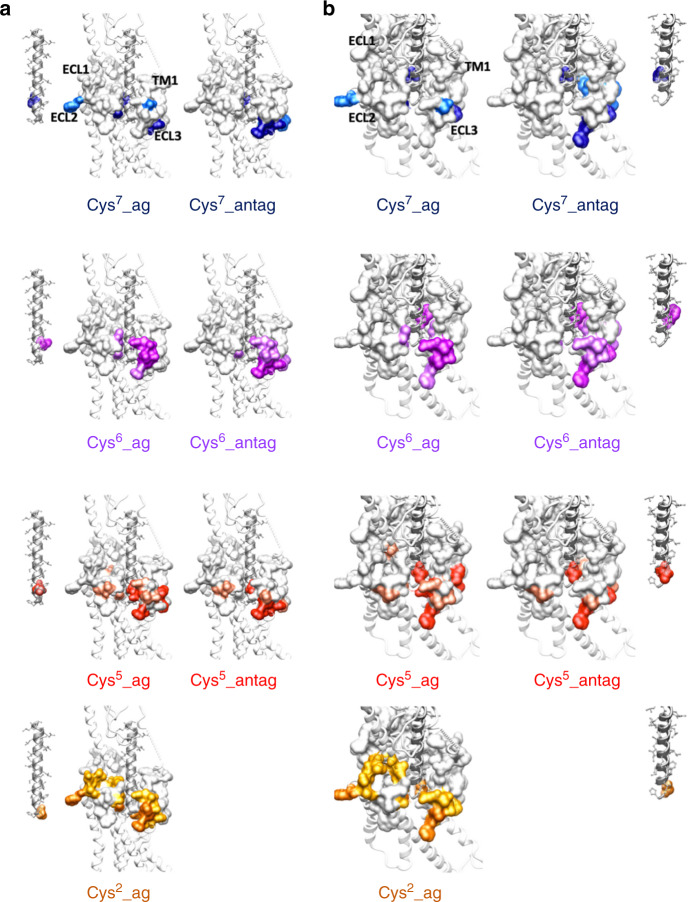
Mapping of cysteine cross-linking data onto the active SecR structure. **a** Side view. Left panel, secretin peptide analog; Middle panel, agonist cross-linking pattern; Right panel, antagonist cross-linking pattern. **b** Magnified view looking into the SecR TM core. Left panel, agonist cross-linking pattern; Middle panel, antagonist cross-linking pattern; Right panel, secretin peptide analog. Peptides are displayed in ribbon format with x-stick representation of side chains, colored gray. The position of cysteine substitution is shown in colored cpk format (Cys^7^, dark blue; Cys^6^, purple; Cys^5^, red; Cys^2^, orange). The SecR is shown in ribbon format with the location of the cysteine mutants displayed in combination cpk and surface representation. Sites of cross-linking are colored according to the site of peptide substitution with dark shading for those with highest efficiency (>50% of the highest efficiency label) and those with intermediate efficiency (25–50% of the highest efficiency label) having light shading.

Disulfide cross-linking between cysteines requires both spatial proximity and appropriate geometry to occur^25^. As such, it has become a useful tool to study trajectories of protein-protein interaction that can include intermediate states involved in peptide binding. We have previously determined the efficiency of the cross-linking of N-terminally Cys-substituted secretin peptide analogs at positions 2, 5, 6, and 7 to SecR with individual Cys mutation throughout the ECLs^23,24^ (Supplementary Table 7), and these can now be mapped onto the active SecR structure. In the current study, we have extended our analysis to include amino acids in the upper segment of TM1, as well as comparison of cross-linking patterns to radiolabelled analogs of the antagonist peptide secretin (c[E^16^, K^20^], I^17^, Cha^22^, R^25^)sec(5–27), cysteine-substituted at positions 5, 6, and 7 (Figs. 5 and 6, Supplementary Tables 8, 9, Supplementary Figs 8–13). No cross-linking was observed within TM1 for any of the agonist analogs (Supplementary Fig. 13). In contrast, cross-linking to Y146^1.43^ was observed for both the Cys^5P^ and Cys^7P^ antagonist peptides (Supplementary Table 9, Supplementary Fig. 13). No cross-linking of antagonist peptides was observed within ECL1 (Supplementary Table 8), consistent with observations for the agonist analogs^23,24^ (Fig. 5). In contrast, extensive cross-linking was observed within ECL2 (Supplementary Fig. 10) and, in particular, ECL3 (Fig. 5A). For the Cys^5P^ analog, efficient cross-linking (>25% cross-linking efficiency relative to the highest signal for any of the peptides) was observed for SecR residues Y146^1.43^, F358^6.56/ECL3^, A359^ECL3^, F360^ECL3^, and S361^ECL3^. The Cys^6P^ and Cys^7P^ also exhibited efficient cross-linking to F358^ECL3^-S361^ECL3^, with the Cys^6P^ also cross-linking efficiently to F372^7.41^ and E373^7.42^. Perhaps unsurprisingly, there was considerable overlap in the location of cross-linking identified for cysteine substitution at equivalent positions in the agonist and antagonist peptides. Nonetheless, important differences occurred in the sites of highest cross-linking efficiency and indeed in the extent of SecR residues that were cross-linked for the two peptides^23,24^ (Supplementary Tables 7–9, Figs. 5, 6). In general, more cross-linking was observed for the agonist substituted peptides that may reflect higher mobility of the receptor bound to the agonist and/or additional conformational sampling due to transducer engagement. In addition, the most efficient site(s) of cross-linking within ECL3 tended to move from the membrane proximal segment of TM6, for the antagonist, to mid-ECL3 or the TM7 proximal segment for the agonist peptides^23,24^ (Fig. 5c), and this is also consistent with ECL3 being the most conformationally divergent among the solved class B GPCR structures^12^.

**Fig. 7:**
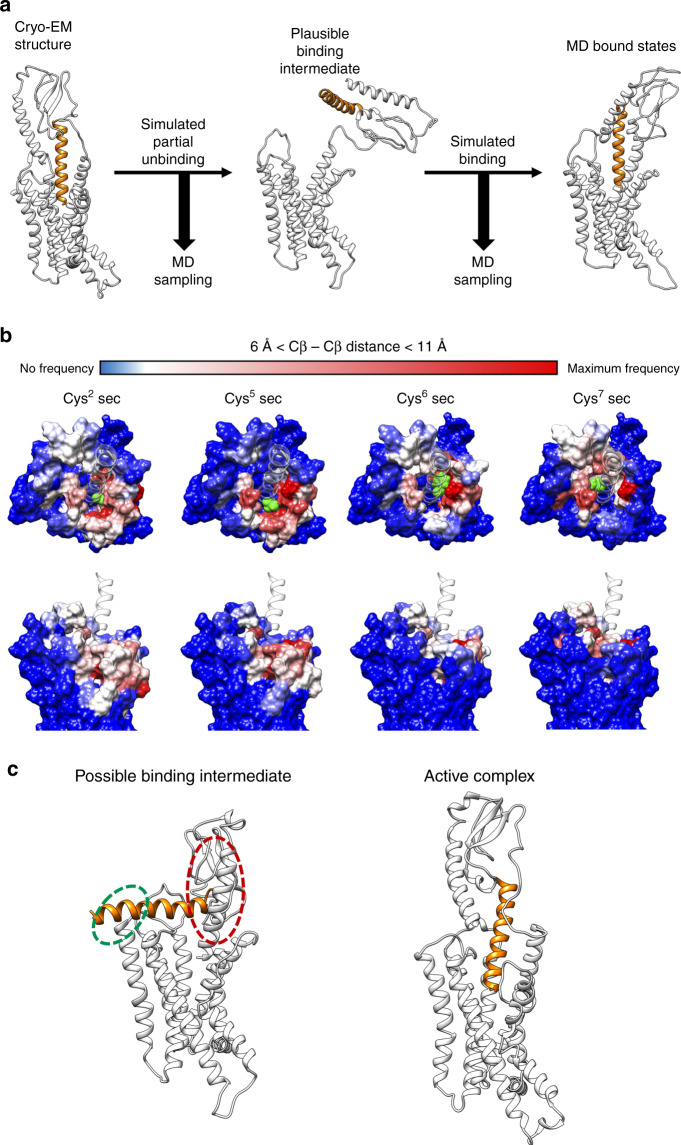
Simulated partial unbinding and binding of secretin using well-tempered metadynamics simulations. **a** Overview of the simulation displayed in Video 3. **b** Heat map of the interaction of secretin residues 2 (left panel), 5 (left middle panel), 6 (right middle panel), and 7 (right panel), equivalent to the positions of cysteine substitution in cross-linking studies, with the SecR core during simulations. The position of the secretin residue in the cryo-EM structure is displayed in green cpk representation, with the rest of the peptide displayed in transparent light gray ribbon format. The receptor core is shown in surface representation colored according to frequency of interactions during the simulation. **c** Speculative schematic illustrating a potential binding intermediate that could account for observed cysteine-cross-linking data prior to the peptide reaching its metastable position observed in the active, G protein-coupled receptor structure.

While cross-linking occurred between the peptide cysteine and receptor residues that were proximal in the active, G protein-bound, structure, in all cases, additional sites of efficient cross-linking were observed, both for agonist and antagonist peptides (Fig. 6). MD simulations to probe partial unbinding and rebinding provided heatmaps for residue proximity that were consistent with most sites of cross-linking identified in the biochemical analysis (Fig. 7a, b, Video 3). However, they did not explain interactions observed for some of the SecR residues in ECL2 (e.g. W295^ECL2^), although this could potentially be accounted for by a binding interaction model where, early in engagement, the peptide C-terminus engaged with the receptor ECD while the peptide was almost horizontal with the membrane (Fig. 7c); this would site the peptide N-terminus in proximity to ECL2 during early phases of binding. Alternatively, the peptide may have a more disordered structure during initial binding allowing simultaneous engagement of the peptide N-terminus and ECL2, and peptide C-terminus and receptor ECD. While speculative, others have previously proposed models of peptide-membrane interaction that could promote peptide secondary structure^26^, potentially providing a mechanistic reason for why this might occur. In this model, the initial peptide engagement would disrupt constraining interactions of the ECD and membrane core to provide the ECD flexibility required to allow key interactions between the peptide N-terminus and the receptor core to form.

### The SecR-G protein interface

**Fig. 8:**
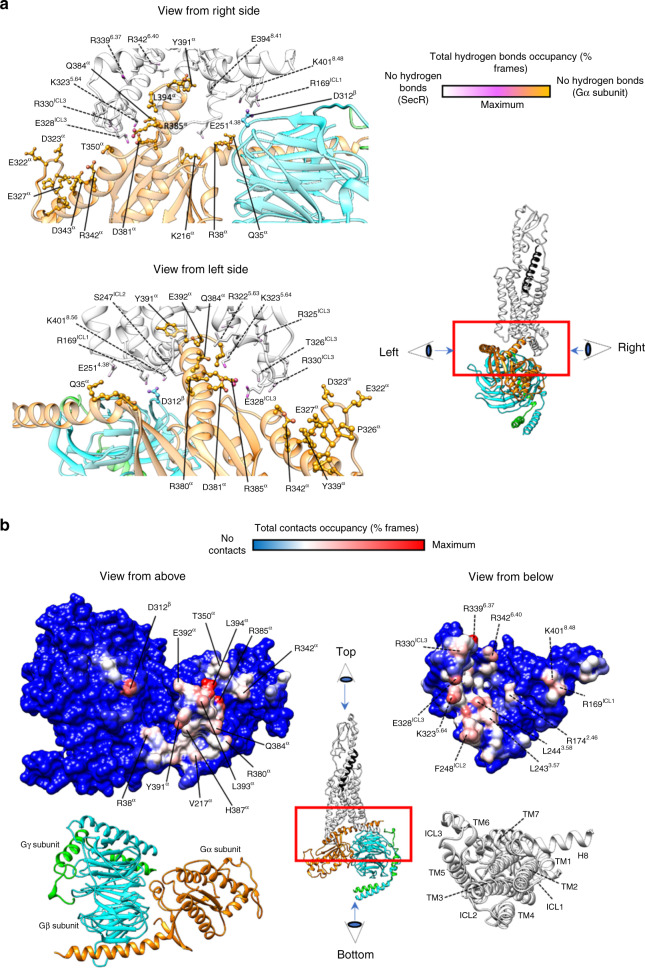
Interactions between SecR and Gs protein during MD simulations. **a** Hydrogen bonds between SecR and Gs protein. The total occupancy (% frames) of each atom is plotted onto the equilibrated complex according to a color scale, with SecR atoms never involved in white, G protein (Gα subunit) never involved in orange, and G protein (Gβ subunit) never involved in cyan; atoms highly involved are magenta. The Gγ subunit is shown as green ribbon, the Gα subunit (ball and stick residues indicated with solid lines) is depicted in orange, partially transparent ribbon, the Gβ subunit (ball and stick residues indicated with solid lines) is shown as cyan, partially transparent ribbon; the SecR (stick residues indicated with dashed lines) is shown as white partially transparent ribbon. The image on the right specifies the relative perspectives from the left and right sides. View from left side) View from the TM6/TM7/TM8 side; View from right side) View from the TM1/TM2/TM3 side. Main hydrogen bonds involved SecR ICL1 (R169^ICL1^), TM5 (K323^5.64^) ICL3 (E328^ICL3^, R330^ICL3^, T326^ICL3^, and R325^ICL3^), TM6 (R339^6.37^ and R342^6.40^), and H8 (K401^8.56^). On the G protein, the α subunit residues L394, Y391, R385, Q384, D381, R342, D343, and Q35 were highly involved; the only β subunit side-chain engaged was D312. **b** Contacts between SecR and Gs protein. The total occupancy (% frames) of each atom is plotted onto the surface of the equilibrated complex according to a color scale, with atoms never involved in blue, and atoms highly involved in red. The central image specifies the relative perspectives from above and below. View from above) Contacts plotted on the G protein surface (the bottom figure shows the ribbon representation as reference). Main interactions involved D312 (Gβ subunit), L394, E392, L393, Y391, H387, R385, Q384, R380, R342, R38 (Gα subunit). View from below) Contacts plotted on the SecR surface (the bottom figure shows the ribbon representation as reference). Main interactions involved ICL1 (R169^ICL1^), TM2 (R174^2.46^), ICL2 (F248^ICL2^), TM3 (L243^3.57^ and L244^3.58^), TM5 (K323^5.64^), ICL3 (E328^ICL3^ and R330^ICL3^), TM6 (R339^6.37^ and R342^6.40^), and H8 (K401^8.56^).

In the static consensus map, SecR forms extensive polar and nonpolar interactions with the G protein, predominantly with the α5-helix of Gαs (Supplementary Table 1, Supplementary Fig 6c, d). As for the secretin:SecR interface, we have used the MD simulations in POPC to interrogate interaction dynamics between SecR and Gs. Not surprisingly, the interactions between the SecR and Gs heterotrimer were similar to those previously reported for other class B GPCRs^12^, with both polar and hydrophobic interactions between the Gαs protein and TMs 2, 3, 5, and 6 (Supplementary Tables 10–12, Fig. 8, Video 4). However, while most other class B GPCRs exhibit contacts between Gαs and the base of TM7 and junction with helix 8 (H8), there were few stable interactions between this domain of the SecR and the G protein. This was most similar to the PTH1R that also lacked substantive interactions with the TM7/H8 junction^12,27^. For the SecR, stable H-bond or salt-bridge interactions were predicted to occur principally between ICL3, and bottoms of TM5 and TM6 and the Gα subunit, with more limited interactions between Q35, R38, Q384 and R380, and ICL2 or the backbone of the nearby L244^3.58^ (Supplementary Tables 10, 11, Fig. 8). The polar interactions with TM5/ICL3/TM6 were predominantly to the α5-helix of Gαs, with more limited interactions predicted to occur with R342 and D323 in the Gα protein. Similar to other class B GPCRs, there were also predicted interactions between the Gβ subunit and the receptor, with the most prominent interactions being between D312^β^ and K401^8.48^ in H8 and R169 in ICL1, which were also observed in the cryo-EM consensus map (Supplementary Table 1, Supplementary Tables 10–12, Fig. 8).

In the SecR EM map, there is clear density for F248^ICL2^ that is located in the junction between the Gs αN helix and α5-helix (Supplementary Fig. 14a). A positionally equivalent aromatic (Phe or Tyr) is seen in most active, G protein-complexed, class B GPCR structures solved to date^12^, as well as the class A Gs-coupled EP2 and β2-adrenergic receptors^28,29^, although select class B GPCRs have a lipophilic Leu, and the GIPR has a distinct sequence^12^. It is hypothesized that interactions between ICL2 and the G protein may contribute to G protein activation, contributing to conformational changes in the G protein linked to GDP release^28^. Interestingly, during the course of the simulations, F248^ICL2^ rapidly exits the αN/α5 junction (Supplementary Fig. 14b, c, Video 4) suggesting that the ICL2-G protein interface is dynamic, and it is possible that the observed orientation of the ICL2 in the consensus map may be partially constrained by the tools used to stabilize the Gα-Gβγ interface (Nb35, DNGαs), although Nb35 was also present in the simulation.

## Discussion

In conclusion, the structure of the SecR provides new insight into peptide binding and activation of class B GPCRs, and the dynamic nature of these interactions. The SecR exhibited the strongest interaction between the receptor ECD and the transmembrane core of the receptor of all class B GPCRs structures solved to date, and this likely contributed to higher relative stability of the ECD relative to the rest of the receptor. This was reflected in the relative robustness of EM density for the ECD, compared to the rest of the receptor, which is less well resolved in other class B GPCR structures. Combining structural data, MD simulations and biochemical cross-linking data advanced understanding of the dynamics of the interaction between secretin and its receptor and models for potential initial engagement of peptide and receptor.

## Methods

### Constructs

Human SecR was modified to include an N-terminal HA tag and FLAG epitope and a C-terminal 8×HIS tag; both of these are removable by 3C protease cleavage. The construct was generated in both mammalian and insect cell expression vectors. Previously described constructs for dominant negative human Gα_s_ (DNGα_s_v1)^9^, (DNGα_s_v2)^10,11^ human His_6_-tagged Gβ_1_, and Gγ_2_^9^ in baculovirus expression vectors were used for complex generation.

### Insect cell expression

The SecR, DNGα_s_v1 or DNGαsv2 (the latter containing an additional A366S mutation)^10^, Gβ_1_, and Gγ_2_ were expressed in *Tni* insect cells (Expression Systems) using baculovirus. For the first preparation, cell cultures were grown in ESF 921 serum-free media (Expression Systems) to a density of 4 million cells/mL, and then infected with three separate baculoviruses at a ratio of 4:2:1 for SecR, DNGα_s_v1, and Gβ_1_γ_2_, respectively. In the second preparation, cell cultures were grown to a density of 3.3 million cells/mL, and then infected with the baculoviruses at a ratio of 3:2:1 for SecR, DNGα_s_v2, and Gβ_1_γ_2_, respectively. Culture was harvested by centrifugation ~48 h post infection and cell pellet was stored at −80 °C.

### Complex purification

Cell pellet was thawed in 20 mM HEPES pH 7.4, 50 mM NaCl, 2 mM MgCl_2_ supplemented with complete Protease Inhibitor Cocktail tablets (Roche) and benzonase nuclease (Merck Millipore). Complex formation was initiated by addition of 1 μM human secretin (China Peptides), Nb35–His (10 μg/mL) and apyrase (25 mU/mL, NEB); the suspension was incubated for 1 h at room temperature. Membranes were collected by centrifugation at 30,000 × *g* for 30 min, and the complex from the membrane was solubilized by 0.5% (w/v) lauryl maltose neopentyl glycol (LMNG, Anatrace) supplemented with 0.03% (w/v) cholesteryl hemisuccinate (CHS, Anatrace) for 2 h at 4 °C in the presence of 1 μM secretin and apyrase (25 mU/mL, NEB). Insoluble material was removed by centrifugation at 30,000 × *g* for 30 min and the solubilized complex was immobilized by batch binding to M1 anti-FLAG-affinity resin in the presence of 3 mM CaCl_2_. The resin was packed into a glass column and washed with 20 column volumes of 20 mM HEPES pH 7.4, 100 mM NaCl, 2 mM MgCl_2_, 3 mM CaCl_2_, 1 μM secretin, 0.01% (w/v) LMNG, and 0.0006% (w/v) CHS before bound material was eluted in buffer containing 5 mM EGTA and 0.1 mg/mL FLAG peptide. The complex was then concentrated using an Amicon Ultra Centrifugal Filter (MWCO 100 kDa) and subjected to SEC on a Superdex 200 Increase 10/300 column (GE Healthcare) that was pre-equilibrated with 20 mM HEPES pH 7.4, 100 mM NaCl, 2 mM MgCl_2_, 1 μM secretin, 0.01% (w/v) MNG, and 0.0006% (w/v) CHS to separate complex from contaminants. Eluted fractions consisting of receptor and G protein complex were pooled and concentrated and stored at −80 °C. Purity and stability of the complex following thawing was confirmed by fSEC. Final yield of purified complex was approximately 0.125 mg/L of insect cell culture.

### SDS–PAGE and western blot analysis

Sample collected from SEC was analyzed by SDS–PAGE and western blot. For SDS–PAGE, precast gradient TGX gels (Bio-Rad) were used. Gels were stained by Instant Blue (Expedeon). Antisera included rabbit anti-Gs C-18 antibody (cat no. sc-383, Santa Cruz), goat anti-rabbit antibody (800CW, LI-COR), mouse Penta-His antibody (cat no. 34660, QIAGEN), and goat anti-mouse antibody (680RD, LI-COR).

### Preparation of vitrified specimen

For the initial preparation, electron microscopy grids (Quantifoil, 200-mesh copper R1.2/1.3) were glow-discharged for 90 s using PELCO easiGlow. Four microliters of sample was applied on the grid in Vitrobot Mark IV chamber (Thermo Fisher Scientific). The chamber of Vitrobot was set to 100% humidity at 4 °C. The sample was blotted for 5.5 s with a blot force of 25, and then plunged into ethane. For the second preparation, acetone prewashed electron microscopy grids (Ultrafoil R1.2/1.3 Au 300 mesh) were glow-discharged and 3 μL of sample was applied to the grid in a Vitrobot Mark IV chamber (Thermo Fisher Scientific), set to 100% humidity at 4 °C. The sample was blotted for 10 s with a blot force of 19, and then flash frozen in liquid ethane.

### Data acquisition

Initial datasets were collected on a Thermo Fisher Scientific Titan Krios microscope operated at 300 kV equipped with a Gatan Quantum energy filter, a Gatan K2 summit direct electron camera (Gatan). Movies were taken in EFTEM nanoprobe mode, with 50-µm C2 aperture and pixel size of 0.86 Å. Each movie comprises 40 subframes with a total dose of 46 e^−^ per Å, with exposure time of 10 s with a dose rate of 3.42 e^−^ pixel^−1^ s^−1^ on the detector. Data acquisition was done using EPU software (Thermo Fisher Scientific) at −500 nm to −1.9 µm defocus (200 nm step). Data from the second sample were collected on a Titan Krios microscope operated at an accelerating voltage of 300 kV with a 50 μm C2 aperture at an indicated magnification of 105,000 × *g* in EFTEM nanoprobe mode and a spot size of 5. A Gatan K3 direct electron detector, positioned post a Gatan Quantum energy filter (Gatan, Pleasanton, CA, USA) with a slit width of 25 eV, was used to collect movies in CDS mode. Movies were recorded as compressed TIFFs in normal-resolution mode yielding a physical pixel size of 0.83 Å/pixel with an exposure time of 5.011 s amounting to a total exposure of 52.9 e^−^/Å2 for at an exposure rate of 7.27 e^−^/pixel/second that was fractionated into 71 subframes. Defocus was varied in the range between −0.7 and −1.5 μm. Beam-image shift, with beam-tilt compensation, was used to acquire data from nine surrounding holes after which the stage was moved to the next collection area using a custom SerialEM script^30,31^.

### Data processing

For the initial sample, a total of 6500 movies were collected and subjected to motion correction using MotionCor2 implemented in RELION v.3.0.7^32^. CTF estimation was done using Gctf software^33^ on nondose-weighted micrographs. The particles were picked using cisTEM^34^. An initial model was made using the common-line approach in EMAN2^35^. Picked particles from cisTEM was imported in RELION v.3.0.7. The particles were extracted using a box size of 240 pixels. A total of 1,200,000 picked particles were subjected to two rounds of 2D classification, followed by 3D classification. After selecting the best-looking class, with 159k particles, 3D auto-refinement was done followed by another round of 3D classification without alignment. Lastly, 77,754 particles from the best-looking class were subjected to 3D auto-refinement.

For the second sample, a total of 5600 movies were collected and subjected to motion correction using MotionCor2^32^ and CTF estimation was done using the Gctf^33^ software on nondose-weighted micrographs, implemented in Relion v3.1-beta^36^. The particles were picked using the automated procedure in crYOLO^37^ and coordinates were imported into Relion. Subsequent data processing steps were carried out using Relion v3.1-beta^36^. Particles were extracted initially using a box of 64 pixels, and after curation of particles in 2D and 3D classifications, re-extracted using a final box size of 288. As an initial 3D reference, a previous GPCR complex map was used and 60 Å low-pass filtered to prevent model bias. 3D references in subsequent steps derived from the data itself. Subsequent rounds of 3D classifications, 3D refinements, and 3D classification without angular and translational alignment were used to create a homogenous set of ~350,000 particles and was further subjected to Bayesian particle polishing and CTF refinements (as implemented in Relion v3.1-beta). A final global 3D refinement using a wide mask (including the entire complex and micelle) was carried out, resulting in a global resolution (FSC = 0.143) of 2.4 Å. In postprocessing, different masks were applied on the global refinement map, resulting in global resolutions (FSC = 0.143) of 2.4 Å using the wide mask and 2.3 Å using a tight mask (excluding the ECD of the receptor, AHD of the Gα protein and micelle) (Fig. 1e–g). To better resolve features of the receptor loops and ECD, the global refinement map was subjected to 3D classification and 3D refinement with fine angular sampling using a mask including only the receptor and peptide (referred to as “receptor-only” refinement). The receptor-only map resulted in a global resolution (FSC = 0.143) of 2.5 Å (Fig. 1f, g). Local resolution estimates and maps were produced in Relion. All masks were created with a custom script using e2proc3D.py from EMAN2^35^.

### Atomic model refinement

Initial models into the 4.3 Å map for SecR complex were made with the Rosetta software package using the structure threading/comparative modeling and model relaxation protocols^38^. Fitting the Rosetta-generated models in the cryo-EM density maps was performed with the MDFF routine in namd2^39^. The fitted models were further refined by rounds of manual model building in coot^40^ and real-space refinement, as implemented in the Phenix software package^41^. The density around the N-terminal ECDs was poorly resolved, and this domain was modeled by further rounds of focused MDFF. As the maps in this region were locally resolved to ~7 Å, only the backbone trace of the protein was kept for deposition. Map and model statistics are detailed in Supplementary Table 13. In the final model (PDB 6WI9), the first modeled residue is R30^ECD^ and the last modeled residue is L408^H8^. Residues R30^ECD^-S130^ECD^ have only been modeled as backbone trace, and amino acids S201^ECL1^-H211^ECL1^ have been omitted from the model.

Based on the initial SecR model (PDB 6WI9), atomic coordinates were refined into the new cryo-EM maps. The majority of the model (Gα, Gβ, Gγ, TMs, and peptide N-terminus) was refined into the global postprocessed map using Phenix^41^ and manually inspected using Coot^40^. Lower resolution areas (in particular ECD, ECL1, and ICL1-3) were furthermore refined using the receptor-only map, initially by flexible fitting and refinement using Isolde^42^, Namdinator^43^ and namd2^39^ and finally by real-space refinement in Phenix and manual inspection in Coot. The density of the ECD remained poorly resolved and therefore only the backbone of the residues R30^ECD^-S130^ECD^ was deposited. The final receptor model (PDB 6WZG) starts with residue R30^ECD^ and ends with residue L408^8.58^ (with residues 30-130^ECD^ of the receptor and 254–263 of the Gα protein modeled as backbone trace). Amino acids from the AHD of the Gα protein (62–204) have been omitted from the model.

### Model residue interaction analysis

Interactions in the PDB (6WZG), between the chains of the peptide and receptor (P:R) or receptor and G proteins (R:A and R:B), were analyzed using the “Dimplot” module within the Ligplot+ program (v2.2)^44^. Hydrogen bonds were additionally analyzed using the UCSF ChimeraX package, with relaxed distance and angle criteria (0.4 Å and 20° tolerance, respectively). Additional analyses and production of images were performed using the UCSF Chimera package (v1.14) from the Computer Graphics Laboratory, University of California, San Francisco (supported by NIH P41 RR-01081) and ChimeraX^45^ (support from National Institutes of Health R01-GM129325).

### 3D variability analysis in Cryosparc

Particle stacks from the Relion global consensus refinement as well as the refinement map were imported into the Cryosparc v2 pipeline^46^. A consensus refinement in Cryosparc using the Homogeneous refinement tool and the imported Relion map as a reference volume was produced, which was used as an input for the 3D variability analysis^11^. For the variability analysis, the wide mask created automatically during refinement in Cryosparc (including micelle) and a 2.8 Å filter was applied. The frames of the three components generated in the 3D variability analysis were visualized in the ChimeraX volume series, and movements were recorded as movies (see also: Supplementary Fig. 15).

### Mammalian cAMP assays

HA-secretin receptor-bearing COS-1 cells (CRL-1650, American Type Culture Collection) were seeded at a density of 30,000 cells/well into 96-well culture plates and incubated over-night in DMEM containing 5% FBS at 37 °C in 5%CO_2_. cAMP detection was performed as previously described^9^. All values were converted to cAMP concentration using a cAMP standard curve performed in parallel and data were subsequently normalized to the response of 100 μM forskolin in each cell line. Data were analyzed by a 3-parameter logistic fit in Prism v7 (GraphPad).

### Peptides

Four cysteine-containing peptides were designed to incorporate a cysteine for disulfide trapping in positions 5, 6, 7, and 10 of the secretin antagonist, (c[E^16^, K^20^], Y^10^, I^17^, Cha^22^, R^25^)sec(5–27) (identified as Cys^5^, Cys^6^-, Cys^7^-, and Cys^10^-antag) that we reported previously^47^. The Cys^5^-antag, Cys^6^-antag, and Cys^7^-antag incorporated a tyrosine to replace the leucine in position 10 for radioiodination^48^, while a tyrosine was incorporated in position 26 of the Cys^10^-antag^24^ (Supplementary Fig. 8a). In addition, five cysteine-containing full-length secretin agonist analogs we synthesized previously^23,24^ were also used in this study for cysteine trapping of the 14 new cysteine mutants of the juxtamembranous region of the amino-terminal domain of the secretin receptor (Supplementary Fig. 8a). All peptides were synthesized and purified by China Peptides (Shanghai, China), with their identities verified by electrospray ionization mass spectrometry.

### Radioiodination

The secretin-like radioligand [Y^10^]secretin(1–27) used in competition ligand binding assays, the newly synthesized Cys^5^-antag, Cys^6^-antag, Cys^7^-antag, and Cys^10^-antag, as well as the five cysteine-containing full-length secretin agonist analogs we synthesized previously^23,24^ were radioiodinated oxidatively using procedures previously described^49^. This was done by incubating ~10 μg of each peptide with 1 mCi Na^125^I in 0.1 M borate buffer (pH 9.0) and exposure for 15 s to the solid phase oxidant, *N*-chlorobenzenesulfonamide (Iodination bead) (Pierce, Rockford, IL). The radioiodinated peptides were purified by reversed-phase HPLC to yield specific radioactivities of ~2000 Ci/mmol using procedures we previously described^49^.

### Receptor constructs

The wild-type human secretin receptor (CHO-SecR) stably expressed in Chinese Hamster Ovary (CHO-K1) cells (CCL-61, American Type Culture Collection) that was established previously^50^ was used for characterizing binding affinities and biological activities of Cys^5^-antag, Cys^6^-antag, Cys^7^-antag, and Cys^10^-antag. This cell line was cultured at 37 °C in an environment containing 5% CO_2_ on tissue culture plasticware in Ham’s F-12 medium supplemented with 5% fetal clone II, and was passaged approximately twice a week.

For cysteine trapping studies with the Cys^5^-antag, Cys^6^-antag, Cys^7^-antag, and Cys^10^-antag probes, wild type and a total of 61 previously characterized secretin receptor constructs incorporating cysteine replacements for natural residues in each of the positions of the three ECLs except for positions with a naturally occurring cysteine were used. They were transiently expressed on COS-1 cells (American Type Culture Collection, Manassas, VA) after transfection using the polyethylenimine method as we have previously described^23^. Cells were maintained in Dulbecco’s modified Eagle’s medium (Invitrogen, Carlsbad, CA) supplemented with 5% fetal clone II and studied 48 h after transfection.

In addition, 14 new constructs incorporating cysteine replacements for natural residues in each of the positions of the juxtamembranous region of the amino-terminal domain of the secretin receptor ranging from residue Lys^113^ to Thr^126^ were generated. These cysteine mutants were prepared using an oligonucleotide-directed approach with the QuikChange site-directed mutagenesis kit from Stratagene (La Jolla, CA) (primers listed in Supplementary Table 14), with the products verified by direct DNA sequencing. They were expressed transiently on COS-1 cells (CRL-1650, American Type Culture Collection) after transfection using a modification of the DEAE-dextran method for binding and biological activity characterization^51^ or using the polyethylenimine method as we have previously described for cysteine trapping experiments^23^. These constructs were used in cysteine trapping studies using both agonist and antagonist cysteine secretin probes.

### Immunostaining

To determine levels of cell surface expression of the 14 new constructs incorporating cysteine replacements for natural residues in each of the positions of the juxtamembranous region of the amino-terminal domain of the secretin receptor, immunostaining using an amino-terminal region secretin receptor antibody was performed in COS-1 cells (CRL-1650, American Type Culture Collection) transiently transfected with these constructs. This polyclonal antibody^52^ was raised by GenScript (Piscataway, NJ) using the peptide antigen representing amino acids 51–65 of the human secretin receptor (anti-hSecR(51–65) that was synthesized and purified in-house.

Transfected cells grown on polylysine-coated glass coverslips in six-well plates for 24 h were washed once with PBS followed by two washes with PBS containing 1% normal goat serum. Coverslips were then incubated for 1 h at room temperature with the anti-hSecR(51–65) polyclonal antibody^52^ (1:500 in PBS with 1% normal goat serum) followed by one PBS wash before being fixed with 2% paraformaldehyde (Electron Microscopy Sciences, Hatfield, PA) in PBS for 15 min. Coverslips were then washed three times with PBS containing 1% normal goat serum and incubated for 1 h with 1:200 Alexa Fluor 488-conjugated anti-rabbit IgG secondary antibody (Molecular Probes, Eugene, OR). Coverslips were washed three times with PBS and mounted on microscope slides with Vectashield mounting medium (Vector Laboratories, Burlingame, CA). All above procedures were performed at room temperature. Cells were visualized with a ×40 objective on a Zeiss inverted microscope controlled by QED In Vivo software (Media Cybernetics, Bethesda, MD). Quantification of receptor cell surface expression as fluorescence percentage of wild-type secretin receptor was done by analyzing 6–8 cells for each of the mutants using the ImageJ software (National Institutes of Health, Bethesda, MD).

### Receptor binding assay

The ability of the Cys^5^-antag, Cys^6^-antag, Cys^7^-antag, and Cys^10^-antag probes to bind to the secretin receptor was assessed by a radioligand competition-binding assay with intact receptor-expressing CHO-SecR cells in 24-well tissue culture plates. In brief, CHO-SecR cells were grown to ~90% confluence and were washed twice with Krebs–Ringers/HEPES (KRH) medium (25 mm HEPES, pH 7.4, 104 mm NaCl, 5 mm KCl, 2 mm CaCl_2_, 1 mm KH_2_PO_4_, 1.2 mm MgSO_4_) containing 0.01% soybean trypsin inhibitor and 0.2% bovine serum albumin. Cells were then incubated with a constant amount of radioligand, ^125^I-[Y^10^]sec(1–27) (~5 pM, ~10,000 cpm) in the absence and presence of increasing concentrations (ranging from 0 to 1 μM) of each of the cysteine-containing secretin antagonist probes for 1 h at room temperature (reaction volume, 250 µL). Cells were then washed twice with ice-cold KRH medium containing 0.01% soybean trypsin inhibitor and 0.2% bovine serum albumin to separate bound from free radioligand before being lysed with 0.5 M NaOH and quantified using a γ-spectrometer. Nonspecific binding was determined in the presence of 0.1 µM unlabeled secretin and represented <15% of total radioligand bound. Binding data were analyzed and plotted using the nonlinear regression analysis program in the Prism software suite version 7.0 (GraphPad Software, San Diego, CA) (Supplementary Fig. 8b). The same assay was also used to characterize the binding activity of COS-1 cells transiently expressing the 14 new receptor cysteine mutant constructs described above.

### Intracellular cAMP assay

The biological activity of the Cys^5^-antag, Cys^6^-antag, Cys^7^-antag, and Cys^10^-antag probes were assessed by examining their ability to stimulate cAMP responses in CHO-SecR cells^50^ using a time-resolved fluorescence-based cAMP assay. In brief, ~8000 cells per well were grown in 96-well plates for 48 h prior to the assay. Cells were washed with PBS and stimulated with increasing concentrations (ranging from 0 to 1 μM) of secretin or each of the cysteine-containing secretin antagonist probes in KRH medium containing 0.01% soybean trypsin inhibitor, 0.2% bovine serum albumin, 0.1% bacitracin, and 1 mM 3-isobutyl-1-methylxanthine for 30 min at 37 °C. After incubation, the reaction solution was aspirated and cells were lyzed with 6% ice-cold perchloric acid for 15 min with vigorous shaking. The cell lysates were adjusted to pH 6 with 30% NaHCO_3_ and assayed for cAMP levels in a 384-well white Optiplate using a LANCE cAMP kit from PerkinElmer (Boston, MA) (Supplementary Fig. 8c). Antagonism of secretin-induced cAMP accumulation was also assessed by co-incubation of increasing concentrations of each of the antagonists with 0.1 nM secretin (Supplementary Fig. 8d). The cAMP concentration-response curves were analyzed and plotted using the nonlinear regression analysis routine in Prism Software Suite (GraphPad, San Diego, CA). The same assay was also used to characterize the biological activity of COS-1 cells transiently expressing the 14 new receptor cysteine mutant constructs described above.

### Cysteine trapping

Four days prior to the cysteine trapping experiments, 5 × 10^4^ COS-1 cells per well were plated in 24-well tissue culture plates. On the following day, cells were transfected in batches with wild type, all the 61 cysteine secretin receptor mutants that we previously used and 14 new receptor cysteine mutant constructs described above using the polyethylenimine method as we have previously described^23^. On the day of assay, medium was removed by aspiration and cells were washed once with DMEM containing 5% fetal clone II before being incubated for 1.5 h at room temperature with 200 μL DMEM containing 5% fetal clone II and ^125^I-Cys^5^-antag, or ^125^I-Cys^6^-antag or ^125^I-Cys^7^-antag or ^125^I-Cys^10^-antag or the five secretin agonist probes we used previously^23,24^ (~100,000 cpm per well) in the absence or presence of 0.1 μM secretin. After the medium was removed, cells were washed once with ice-cold PBS and incubated with 80 µL SDS Laemmli sample buffer with or without 0.1 M dithiothreitol on a shaker for 45 min. Cells were then scraped and the lysates were transferred to 1.5 mL microcentrifuge tubes using wide-bore tips. Samples were briefly sonicated to break the sticky DNA and resolved in 10% SDS-polyacrylamide gels. Gels were dried, and bands of interest were visualized by autoradiography with band densitometry analyzed by the ImageJ software. The apparent molecular weights of the radioactive bands were determined by interpolation on a plot of the mobility of the appropriate ProSieve protein markers (Cambrex, Rockland, ME, USA) vs. the log values of their apparent masses.

### Modeling methods

The MD was based on the initial PDB model (6WI9) that was available at the time. The missing stalk region was generated using Modeller^53^ and refined using the loop modeling feature; the loop with the lowest DOPE score^54^ (out of 2000 generated loops) was selected. The missing loops in the G protein (L296-K307, C365-E370) were generated from the corresponding loops in the β_2_-adrenergic receptor–G protein complex, PDB code 3SN6^55^ by fitting them with VMD^56^ to the flanking residues. The 3SN6 G protein X-ray structure is 99% identical to the G protein used in this study; it generally gave a lower root mean squared deviation value on molecular superposition than the alternatives (e.g., PDB code 6b3j). The joining point was taken as the closest atom pairs (usually separated by ~0.2 Å) that maintained an appropriate Cα–Cα distance (3.8 ± 2 Å) across the join; selected residues spanning the join were minimized using PLOP^57^ where additional refinement was desirable. The S250–T263 loop was completed using the shorter loop from the adenosine A2A receptor–G-protein complex, PDB code 5G53^58^. The helical domain (residues A48—V204) was not visible in the cryo-EM structure and was omitted as in earlier work^12^.

### Systems preparation

The full-length model of the secretin:SecR:G protein:Nb35 complex was prepared for simulation with the CHARMM36 force field^59^, through use of in-house python htmd^60^ and TCL (Tool Command Language) scripts. The pdb2pqr^61^ and propka^62^ software were used to add hydrogen atoms appropriate for a simulated pH of 7.0; the protonation of titratable side chains was checked by visual inspection. The obtained structure was superimposed on the GLP-1R (PDB ID 5VAI) from the OPM database^63^ so as to orient the receptor prior to insertion in a rectangular prebuilt 125 Å × 116 Å 1-palmitoyl-2-oleyl-sn-glycerol-3-phosphocholine (POPC) bilayer; lipid molecules overlapping the receptor were removed. TIP3P water molecules were added to the 125 Å × 116 Å × 178 Å simulation box using the VMD Solvate plugin 1.5 (Solvate Plugin, Version 1.5. at <http://www.ks.uiuc.edu/Research/vmd/plugins/solvate/). Overall charge neutrality was maintained by adding Na^+^ and Cl^−^ counter ions to a final ionic concentration of 150 mM using the VMD Autoionize plugin 1.3 (Autoionize Plugin, Version 1.3. at <http://www.ks.uiuc.edu/Research/vmd/plugins/autoionize/).

### Systems equilibration and MD settings

ACEMD^64^ was used for both equilibration and MD productive simulations. Isothermal-isobaric conditions (Langevin thermostat^65^ with a target temperature of 300 K and damping of 1 ps^−1^ and Berendsen barostat^66^ with a target pressure 1 atm) were employed to equilibrates the systems through a multistage procedure (integration time step of 2 fs). Initial steric clashes between lipid atoms were reduced through 2500 conjugate-gradient minimization steps, then a 2 ns MD simulation was run with a positional constraint of 1 kcal mol^−1^ Å^−2^ on protein atoms and lipid phosphorus atoms. Subsequently, 20 ns of MD were performed constraining only the protein atoms. In the final equilibration stage, only the protein backbone alpha carbons were constrained, to a total simulation time of 100 ns.

Productive trajectories in the canonical ensemble (NVT) at 300 K (three replicas of 0.94 μs, 0.88 μs, and 1.00 μs, respectively) were computed using a thermostat damping of 0.1 ps^−1^ with an integration time step of 4 fs and the M-SHAKE algorithm^67^ to constrain the bond lengths involving hydrogen atoms. The cut-off distance for electrostatic interactions was set at 9 Å, with a switching function applied beyond 7.5 Å. Long range Coulomb interactions were handled using the particle mesh Ewald summation method^53^ (PME) by setting the mesh spacing to 1.0 Å. Trajectory frames were written every 100 ps of simulations.

### Secretin partial unbinding and binding protocol

The secretin:SecR complex (obtained removing both the G protein and Nb35) was prepared and equilibrated as reported above. The secretin partial unbinding was then simulated in two different well-tempered metadynamics replicas^68^, biasing the distance between the residues H1-R14 (secretin) and K135^1.31^-L391^7.60^ (SecR) centroids. Plumed2.3^69^ was used to seed a Gaussian energy function every 1 ps (height = 0.1 kcal/mol, width = 0.1 Å, with a biasfactor = 20), at a simulated temperature of 300 K, until the distance reached 30 Å.

Starting from the final state of one of the two unbinding trajectories, the secretin partial binding was simulated in six replicas supervising^70^ the same centroids distance considered for the partial unbinding, during successive time windows of 2 ns.

To increase the overall MD sampling, each unbinding/binding replica was grouped into 1 Å width bins according to the root mean square fluctuation (RMSF) of the secretin Cα carbon to the initial positions. One frame was extracted from each cluster and used as seed for a 30 ns long classic MD simulation, for a resulting total MD time sampling of 6.4 μs.

### MD analysis

Atomic contacts were computed using the GetContacts analysis tool (at https://getcontacts.github.io/), with the donor-acceptor threshold distance set to 3.5 Å and the angle set to 120°. Videos were generated using VMD^56^ and avconv (at https://libav.org/avconv.html). Root mean square deviation and RMSF values were computed using VMD^56^ after superposition of the MD trajectories frames on the alpha carbon of the TM domain (residues K134^1.31^ to L391^7.60^). VMD was also employed to compute the Cβ–Cβ carbon distances on the 6.4 μs nonequilibrium MD (secretin partial unbinding/binding), selecting the Cβ carbon of secretin residues S2, T5, F6, and T7 and all the SecR Cβ atoms.

### Reporting summary

Further information on research design is available in the Nature Research Reporting Summary linked to this article.

## Supplementary information

Supplementary information

Video 1

Video 2

Video 3

Video 4

Reporting Summary

## Data Availability

Data supporting the findings of this manuscript are available from the corresponding authors upon reasonable request. A reporting summary for this article is available as a Supplementary Information file. Atomic coordinates and the cryo-EM density map have been deposited in the Protein Data Bank (PDB) under accession numbers PDB 6WI9 (low resolution model) and PDB 6WZG (high-resolution model), and Electron Microscopy Data Bank (EMDB) accession numbers EMD-21683 (4.3 Å maps) and EMD-21972 (high-resolution maps). Source data are provided with this paper.

## Source data

Source Data File
