# Supplementary material for: Structure and dynamics of the active Gs-coupled human secretin receptor
Source: Nat Commun. 2020 Aug 18;11:4137. doi: 10.1038/s41467-020-17791-4 (PMC7435274; doi:10.1038/s41467-020-17791-4)
Supplement: Supplementary file 1 — Supplementary information [file 41467_2020_17791_MOESM1_ESM.pdf]

**Structure and dynamics of the active, Gs-coupled, human secretin receptor**

Maoqing Dong[1*], Giuseppe Deganutti[2*], Sarah J. Piper[3*],Yi-Lynn Liang[3*], Maryam Khoshouei[4*^], Matthew J. Belousoff[3], Kaleeckal G. Harikumar[1], Christopher A Reynolds[2], Alisa Glukhova[3], Sebastian G. B. Furness[3],Arthur Christopoulos[3], Radostin Danev[5], Denise Wootten[3#], Patrick M. Sexton[3#], Laurence J. Miller[1#]

**Supplementary Information**

Supplementary Tables 1 –14. Pages 1 – 12.
Supplementary Figures 1 –15. Pages 13 - 27

**Supplementary Table 1.** SecR interaction with secretin peptide and G protein subunits derived from PDB 6WZG. LigPlot+ was used to identify interacting residues. Only secretin residues 1-23 were used to calculate interactions due to the limited resolution in the receptor ECD. Both side-chain and backbone H-bond interactions are included, and displayed in bold type.

| Secretin | SecR | Interaction | Gαs | SecR | Interaction |
|---|---|---|---|---|---|
| **H1** | **Q223$^{3.37}$** | **H-bond** | H41 | F248$^{ICL2}$ | hydrophobic |
| H1 | I226$^{3.40}$ | hydrophobic | V217 | F248$^{ICL2}$ | hydrophobic |
| H1 | I298$^{5.44}$ | hydrophobic | F219 | F248$^{ICL2}$ | hydrophobic |
| H1 | Y230$^{3.44}$ | hydrophobic | Y358 | Q327$^{ICL3}$ | hydrophobic |
| H1 | W295$^{5.36}$ | hydrophobic | F376 | F248$^{ICL2}$ | hydrophobic |
| **S2** | **E373$^{7.42}$** | **H-bond** | C379 | F248$^{ICL2}$ | hydrophobic |
| S2 | L370$^{7.39}$ | hydrophobic | **R380** | **A245$^{ICL2}$** | **H-bond** |
| S2 | Q369$^{ECL3}$ | hydrophobic | **R380** | **S247$^{ICL2}$** | **H-bond** |
| **D3** | **Y150$^{1.47}$** | **H-bond** | R380 | F248$^{ICL2}$ | hydrophobic |
| **D3** | **R188$^{2.60}$** | **H-bond** | **D381** | **K323$^{5.64}$** | **H-bond** |
| **D3** | **N192$^{2.64}$** | **H-bond** | **Q384** | **K323$^{5.64}$** | **H-bond** |
| D3 | L374$^{7.43}$ | hydrophobic | **Q384** | **L244$^{ICL2}$** | **H-bond** |
| D3 | F222$^{3.36}$ | hydrophobic | **R385** | **T326$^{ICL3}$** | **H-bond** |
| F6 | L374$^{7.43}$ | hydrophobic | R385 | K323$^{5.64}$ | hydrophobic |
| F6 | L139$^{1.36}$ | hydrophobic | H387 | L243$^{3.57}$ | hydrophobic |
| F6 | K143$^{1.40}$ | hydrophobic | H387 | S250$^{ICL2}$ | hydrophobic |
| F6 | Y146$^{1.43}$ | hydrophobic | L388 | L244$^{ICL2}$ | hydrophobic |
| **T7** | **K195$^{2.67}$** | **H-bond** | Q390 | R174$^{2.46}$ | hydrophobic |
| T7 | F222$^{3.36}$ | hydrophobic | Y391 | Y239$^{3.53}$ | hydrophobic |
| T7 | F200$^{ECL1}$ | hydrophobic | Y391 | R174$^{2.46}$ | hydrophobic |
| **S8** | **N289$^{ECL2}$** | **H-bond** | Y391 | L240$^{3.54}$ | hydrophobic |
| S8 | I288$^{ECL2}$ | hydrophobic | **E392** | **G393$^{8.40}$** | **H-bond** |
| S8 | D287$^{ECL2}$ | hydrophobic | **E392** | **R342$^{6.40}$** | **H-bond** |
| **E9** | **R135$^{1.32}$** | **H-bond** | **L393** | **S343$^{6.41}$** | **H-bond** |
| E9 | M366$^{7.35}$ | hydrophobic | E392 | N392$^{7.61}$ | hydrophobic |
| L10 | K143$^{1.40}$ | hydrophobic | L393 | R339$^{6.37}$ | hydrophobic |
| L10 | L139$^{1.36}$ | hydrophobic | L394 | R339$^{6.37}$ | hydrophobic |

| | | |
|---|---|---|
| **S11** | **D287$^{ECL2}$** | **H-bond** |
| S11 | F200$^{ECL1}$ | hydrophobic |
| S11 | I288$^{ECL2}$ | hydrophobic |
| R12 | I288$^{ECL2}$ | hydrophobic |
| L13 | H136$^{1.33}$ | hydrophobic |
| L13 | L139$^{1.36}$ | hydrophobic |
| **R14** | **S202$^{ECL1}$** | **H-bond** |
| R14 | H136$^{1.33}$ | hydrophobic |
| R14 | F200$^{ECL1}$ | hydrophobic |
| **E15** | **L31$^{ECD}$** | **H-bond** |
| E15 | R30$^{ECD}$ | hydrophobic |
| **R18** | **D203$^{ECL1}$** | **H-bond** |
| R18 | V205$^{ECL1}$ | hydrophobic |
| **R21** | **D203$^{ECL1}$** | **H-bond** |
| L23 | V125$^{1.22}$ | hydrophobic |

| | | |
|---|---|---|
| L394 | L320$^{5.61}$ | hydrophobic |

| Gβ | SecR | Interaction |
|---|---|---|
| **D312** | **K401$^{8.48}$** | **H-bond** |
| D312 | R169$^{ICL1}$ | hydrophobic |
| R52 | R168$^{ICL1}$ | hydrophobic |

**Supplementary Table 2.** Main SecR:secretin hydrogen bonds (side chain-side chain) during MD simulations. Data are expressed as the occupancy (% of frames) in which the interactions were present.

| Secretin receptor residue | Secretin residue | Occupancy (%frames) |
|---|---|---|
| **R135$^{1.32}$** | E9 | 83.7 |
| **R30$^{ECD}$** | E15 | 73.9 |
| **E373$^{7.42}$** | S2 | 68.7 |
| **R188$^{2.60}$** | D3 | 51.5 |
| **R299$^{5.40}$** | D3 | 31.1 |
| **D204$^{ECL1}$** | R21 | 31.0 |
| **D203 $^{ECL1}$** | R18 | 30.9 |
| **D204 $^{ECL1}$** | R21 | 30.5 |
| **D203 $^{ECL1}$** | R18 | 27.8 |
| **Y146$^{1.43}$** | D3 | 26.8 |
| **D209 $^{ECL1}$** | R18 | 25.6 |
| **D209 $^{ECL1}$** | R18 | 22.3 |
| **D203 $^{ECL1}$** | R14 | 17.6 |
| **D203 $^{ECL1}$** | R14 | 16.5 |
| **D203 $^{ECL1}$** | R21 | 14.8 |
| **D204 $^{ECL1}$** | R18 | 14.0 |
| **R299$^{5.40}$** | H1 | 13.9 |
| **D203 $^{ECL1}$** | R21 | 13.6 |
| **D196$^{2.68}$** | R14 | 13.4 |
| **D196$^{2.68}$** | R14 | 12.9 |
| **D204 $^{ECL1}$** | R18 | 12.4 |
| **Y230$^{3.44}$** | H1 | 10.4 |
| **N289$^{ECL2}$** | T5 | 10.1 |
| **D209 $^{ECL1}$** | R14 | 9.4 |

| | | |
|---|---|---|
| **D209$^{ECL1}$** | R14 | 8.1 |
| **N120$^{ECD}$** | Q24 | 7.2 |
| **Q223$^{3.37}$** | H1 | 6.7 |

**Supplementary Table 3.** Main SecR:secretin hydrogen bonds (side chain-backbone) during MD simulations. Data are expressed as the occupancy (% of frames) in which the interactions were present. (bb) indicates residue involved at the backbone atoms level (if both of the two residues are indicated with bb, then alternated side chain-backbone interactions occurred during MD).

| Secretin receptor residue | Secretin residue | Occupancy (%frames) |
|---|---|---|
| **E373$^{7.42}$** | H1(bb) | 89.7 |
| **E373$^{7.42}$** | S2(bb) | 58.9 |
| **N289$^{ECL2}$(bb)** | S8 | 50.6 |
| **N72$^{ECD}$** | L26(bb) | 41.1 |
| **R30$^{ECD}$(bb)** | E15 | 28.0 |
| **D203$^{ECL1}$(bb)** | R18 | 21.9 |
| **R299$^{5.40}$** | H1(bb) | 14.6 |
| **S201$^{ECD}$(bb)** | R14 | 14.1 |
| **A365$^{ECL3}$(bb)** | T5 | 13.7 |
| **N289$^{ECL2}$** | G4(bb) | 11.6 |
| **D203$^{ECL1}$(bb)** | R21 | 11.3 |
| **L199$^{2.71}$(bb)** | R14 | 9.8 |
| **E363$^{ECL3}$(bb)** | H1(bb) | 8.2 |
| **L199$^{2.71}$(bb)** | R18 | 8.2 |
| **N72$^{ECD}$** | V27(bb) | 6.0 |
| **D204$^{ECL1}$(bb)** | R18 | 6.0 |
| **D287$^{ECL2}$(bb)** | T7 | 5.6 |
| **M366$^{7.35}$(bb)** | H1 | 5.6 |

**Supplementary Table 4.** SecR:secretin generic contacts during MD simulations. Contacts are determined as the occupancy (% of frames) in which at least two atoms were in contact.

| Secretin receptor residue | Secretin residue | Occupancy (%frames) |
|---|---|---|
| **E373$^{7.42}$** | H1 | 94.0 |
| **E373$^{7.42}$** | S2 | 86.5 |
| **H136$^{1.33}$** | L13 | 86.3 |
| **R135$^{1.32}$** | E9 | 84.3 |
| **R30$^{ECD}$** | E15 | 82.6 |
| **L374$^{7.43}$** | F6 | 74.5 |
| **N72$^{ECD}$** | L26 | 72.9 |
| **V205$^{ECL1}$** | R18 | 61.0 |
| **L96$^{ECD}$** | L19 | 60.8 |

| | | |
|---|---|---|
| **I288 $^{ECL2}$** | S8 | 60.2 |
| **L199$^{2.71}$** | R14 | 60.1 |
| **N289 $^{ECL2}$** | S8 | 59.6 |
| **L370$^{7.39}$** | S2 | 59.1 |
| **L139$^{1.36}$** | E9 | 54.8 |
| **D203 $^{ECL1}$** | R18 | 54.8 |
| **R188$^{2.60}$** | D3 | 52.3 |
| **L142$^{1.39}$** | F6 | 50.2 |
| **L28 $^{ECD}$** | E15 | 50.1 |
| **L31 $^{ECD}$** | L19 | 49.4 |
| **L38 $^{ECD}$** | L26 | 45.6 |
| **K143$^{1.40}$** | L10 | 45.2 |
| **D287 $^{ECL2}$** | S11 | 43.2 |
| **L31 $^{ECD}$** | R18 | 43.0 |
| **K143$^{1.40}$** | F6 | 42.5 |
| **I288 $^{ECL2}$** | S11 | 41.9 |
| **Y146$^{1.43}$** | F6 | 41.6 |
| **L96 $^{ECD}$** | L23 | 41.0 |
| **I288 $^{ECL2}$** | R12 | 41.0 |
| **D287 $^{ECL2}$** | S8 | 39.5 |
| **V34 $^{ECD}$** | L19 | 39.1 |
| **R299$^{5.40}$** | H1 | 37.7 |
| **F92 $^{ECD}$** | L23 | 37.5 |
| **V205 $^{ECL1}$** | L22 | 37.4 |
| **N289 $^{ECL2}$** | G4 | 36.7 |
| **L139$^{1.36}$** | F6 | 35.9 |
| **I73 $^{ECD}$** | L26 | 34.3 |
| **F200 $^{ECL1}$** | R14 | 33.5 |
| **I73 $^{ECD}$** | L23 | 33.3 |
| **V205 $^{ECL1}$** | R21 | 32.9 |
| **F92 $^{ECD}$** | L26 | 32.8 |
| **L139$^{1.36}$** | L13 | 32.2 |
| **D204 $^{ECL1}$** | R21 | 32.2 |
| **R30 $^{ECD}$** | R12 | 32.1 |
| **R299$^{5.40}$** | D3 | 32.1 |
| **L96 $^{ECD}$** | Q20 | 32.0 |
| **I73 $^{ECD}$** | V27 | 31.9 |
| **V34 $^{ECD}$** | L22 | 31.6 |
| **F358$^{6.56}$** | H1 | 31.3 |
| **W295$^{5.36}$** | H1 | 30.3 |

**Supplementary Table 5#.** Secretin binding affinity and cAMP response for wild type and extracellular loop cysteine mutant secretin receptors expressed in COS-1 cells.

| Receptor constructs | Secretin binding | | Intracellular cAMP response | |
|---|---|---|---|---|
| | $K_i$ nM | $B_{max}$ binding sites/cell ×$10^3$ | $EC_{50}$ nM | $E_{max}$ pmol/$10^6$ cells |
| WT | 1.2 ± 0.1 | 111.7 ± 4.9 | 0.02 ± 0.01 | 189 ± 46 |
| **ECL1** | | | | |
| D196C | N.D. | N.D. | 566 ± 58** | 156 ± 44 |
| A197C | 8.3 ± 1.2** | 101.3 ± 46.6 | 0.04 ± 0.01 | 167 ± 56 |
| V198C | 2.7 ± 0.2** | 4.3 ± 1.0** | 0.4 ± 0.1* | 166 ± 39 |
| L199C | N.D. | N.D. | 33.2 ± 5.6** | 159 ± 50 |
| F200C | N.D. | N.D. | 16.2 ± 4.2* | 178 ± 40 |
| S201C | N.D. | N.D. | 1.5 ± 0.5* | 158 ± 58 |
| S202C | N.D. | N.D. | 6.3 ± 1.9* | 176 ± 45 |
| D203C | N.D. | N.D. | 3.9 ± 0.5** | 156 ± 54 |
| D204C | N.D. | N.D. | 2.1 ± 0.5* | 189 ± 47 |
| V205C | N.D. | N.D. | 2.1 ± 0.4** | 176 ± 38 |
| T206C | 1.9 ± 0.4 | 9.8 ± 3.9** | 0.3 ± 0.1* | 167 ± 36 |
| Y207C | N.D. | N.D. | 0.3 ± 0.05** | 156 ± 48 |
| D209C | 2.7 ± 0.2** | 6.9 ± 3.4** | 0.2 ± 0.07 | 190 ± 46 |
| A210C | 3.9 ± 0.7* | 4.8 ± 1.4** | 2.2 ± 0.7* | 178 ± 34 |
| H211C | N.D. | N.D. | 2.1 ± 0.4** | 176 ± 55 |
| R212C | N.D. | N.D. | 4.1 ± 1.2* | 165 ± 34 |
| A213C | 5.4 ± 0.7** | 19.2 ± 5.3* | 0.1 ± 0.04 | 202 ± 59 |
| G214C | 4.8 ± 0.8* | 22.1 ± 6.8** | 0.08 ± 0.03 | 201 ± 38 |
| K216C | 5.4 ± 0.4** | 7.9 ± 1.3** | 0.3 ± 0.1* | 198 ± 49 |
| L217C | 7.1 ± 0.5** | 62.0 ± 11.7* | 0.02 ± 0.01 | 200 ± 25 |
| V218C | 6.6 ± 0.3** | 53.5 ± 4.7** | 0.04 ± 0.01 | 166 ± 36 |
| M219C | 3.5 ± 0.6* | 4.1 ± 0.5** | 0.3 ± 0.1* | 159 ± 34 |
| V220C | 8.5 ± 1.4** | 67 ± 0.6** | 0.04 ± 0.02 | 169 ± 45 |
| L221C | 6.9 ± 0.5** | 57.7 ± 21.4 | 0.02 ± 0.004 | 168 ± 48 |
| F222C | 19.7 ± 2.3** | 55.9 ± 12.6* | 1.5 ± 0.6 | 166 ± 48 |
| Q223C | 8.2 ± 1.5** | 22.4 ± 9.1** | 1.2 ± 0.4* | 191 ± 23 |
| **ECL2** | | | | |
| F279C | 9 ± 1.8* | 89.7 ± 15.0 | 0.04 ± 0.01 | 209 ± 65 |
| L280C | 6.8 ± 1.2** | 69.7 ± 19.7 | 0.06 ± 0.01* | 187 ± 54 |
| E281C | 3.3 ± 0.4** | 2.6 ± 0.1** | 4.4 ± 1.2* | 157 ± 43 |
| D282C | N.D. | N.D. | 0.3 ± 0.1* | 199 ± 50 |
| V283C | 2.7 ± 0.7 | 6.9 ± 0.9** | 0.2 ± 0.04* | 167 ± 39 |
| G284C | N.D. | N.D. | 3.4 ± 1.1* | 155 ± 55 |
| W286C | N.D. | N.D. | N.D. | N.D. |
| D287C | 2.5 ± 0.2** | 2.8 ± 0.4** | 0.8 ± 0.2* | 177 ± 40 |
| I288C | 7.9 ± 1.9* | 23.3 ± 7.1** | 0.5 ± 0.2 | 197 ± 42 |
| N289C | 5.9 ± 1.3* | 23.1± 2.3** | 0.3 ± 0.1* | 178 ± 37 |
| A290C | 3.4 ± 0.2** | 10.6 ± 1.1** | 0.1 ± 0.04 | 177 ± 36 |
| N291C | 6.5 ± 0.3** | 45.8 ± 7.4** | 0.03 ± 0.01 | 197 ± 29 |
| A292C | 5.5 ± 0.7** | 43.3 ± 6.1** | 0.07 ± 0.03 | 170 ± 57 |
| S293C | 9.3 ± 1.7** | 52.5 ± 17.9* | 0.1 ± 0.03 | 176 ± 43 |
| I294C | 9.2 ± 0.7** | 40.9 ± 17.5* | 0.2 ± 0.05* | 188 ± 35 |
| W295C | 28.9 ± 3.8** | 46.3 ± 12.8** | 3.4 ± 0.8* | 187 ± 41 |
| W296C | 6.4 ± 0.8** | 33.0 ± 13.4** | 0.05 ± 0.02 | 205 ± 33 |
| I297C | 5.5 ± 0.7** | 35.9 ± 16.3* | 0.04 ± 0.02 | 196 ± 36 |
| **ECL3** | | | | |
| F358C | 23.1 ± 3.0** | 88.6 ± 26.3 | 3.2 ± 0.9* | 156 ± 46 |
| A359C | 7.6 ± 0.6** | 53.4 ± 17.8 | 0.07 ± 0.03 | 175 ± 55 |
| F360C | 10.0 ± 1.3** | 44.8 ± 20.5* | 0.1 ± 0.02* | 185 ± 45 |
| S361C | 12.3 ± 1.0** | 63.8 ± 21.3 | 0.05 ± 0.02 | 187 ± 49 |
| P362C | 5.1 ± 0.8** | 24.1 ± 8.7** | 0.4 ± 0.1* | 199 ± 40 |
| E363C | 4.0 ± 0.6* | 12.5 ± 5.0** | 0.1 ± 0.03 | 212 ± 52 |
| D364C | 5.2 ± 0.6** | 21.87 ± 7.1** | 0.06 ± 0.02 | 200 ± 56 |
| A365C | 9.2 ± 1.9* | 35.51 ± 12.0** | 0.06 ± 0.03 | 181 ± 41 |
| M366C | 7.2 ± 0.9** | 18.1 ± 5.6** | 0.3 ± 0.1* | 182 ± 36 |
| E367C | 5.3 ± 1.0* | 12.6 ± 5.1** | 0.3 ± 0.04** | 197 ± 47 |
| I368C | 5.0 ± 0.6** | 27.7 ± 7.3** | 0.1 ± 0.04 | 202 ± 42 |
| Q369C | 5.0 ± 0.7** | 37.4 ± 15.1** | 0.05 ± 0.01 | 204 ± 52 |
| L370C | 6.2 ± 0.9** | 11.9 ± 2.8** | 0.2 ± 0.04* | 179 ± 46 |
| F371C | 3.7 ± 0.5 | 26.5 ± 13.4** | 0.03 ± 0.01 | 170 ± 39 |
| F372C | 11.0 ± 0.9** | 36.8 ± 19.4* | 0.2 ± 0.03** | 169 ± 50 |
| E373C | 6.8 ± 1.5* | 27.5 ± 7.8** | 0.5 ± 0.2 | 194 ± 44 |
| L374C | 4.9 ± 0.3** | 87.0 ± 23.9 | 1.0 ± 0.2** | 210 ± 42 |

**#**Data are from reference #23, and re-tabled with SecR residues number from the initiator methionine as residue 1 to assist in data comparisons within the current manuscript.

**Supplementary Table 6.** Secretin binding affinity and cAMP response for wild type and TM1/juxtamembranous region cysteine mutant secretin receptors expressed in COS-1 cells.

| Receptor constructs | Secretin binding | | Intracellular cAMP response to secretin |
|---|---|---|---|
| | ***pK*~i~** → $pK_i$ | $B_{max}$ x$10^5$**sites/cell** | $pEC_{50}$ |
| WT | 8.7 ± 0.1 | 0.8 ± 0.1 | 10.8 ± 0.2 |
| K134C | 9.4 ± 0.1 | 0.6 ± 0.1 | 11.2 ± 0.3 |
| R135C | 8.9 ± 0.2 | 0.6 ± 0.1 | 9.9 ± 0.3 |
| H136C | 9.3 ± 0.3 | 0.6 ± 0.05 | 10.1 ± 0.4 |
| S137C | 9.6 ± 0.2* | 0.6 ± 0.1 | 10.4 ± 0.2 |
| Y138C | 9.4 ± 0.2 | 0.6 ± 0.1 | 10.7 ± 0.2 |
| L139C | *N.D.* | *N.D.* | 8.8 ± 0.06* |
| L140C | 8.6 ± 0.1 | 0.7 ± 0.1 | 10.3 ± 0.3 |
| K141C | *N.D.* | *N.D.* | 9.5 ± 0.2* |
| L142C | 8.8 ± 0.3 | 0.8 ± 0.1 | 10.9 ± 0.1 |
| K143C | *N.D.* | *N.D.* | 9.6 ± 0.1 |
| V144C | 8.9 ± 0.1 | 0.8 ± 0.1 | 10.9 ± 0.6 |
| M145C | 8.6 ± 0.3 | 0.7 ± 0.1 | 10.5 ± 0.2 |
| Y146C | 8.1 ± 0.1* | 0.7 ± 0.1 | 9.3 ± 0.4* |
| T147C | 8.6 ± 0.3 | 0.7 ± 0.1 | 9.9 ± 0.4 |

Shown are $pK_i$ and $B_{max}$ values of secretin binding to COS-1 cells expressing each of the noted secretin receptor constructs. Shown also are $EC_{50}$ values of secretin-stimulated intracellular cAMP accumulation in these cells. All values represent means ± SEM of data from a minimum three independent experiments performed in duplicate. Asterisk denotes that the value is significantly different from that of wild type (WT) secretin receptor determined using ANOVA with Dunnett's post-test ($p<0.05$). *N.D.*, binding not detectable.

**Supplementary Table 7.** Summary of the highest efficiency cysteine trapping constraints (human SecR sequence) by cysteine secretin agonist probes.

| Secretin peptide residue | Secretin receptor residues | References |
|---|---|---|
| $Cys^2$ | $Phe^{279}$, $Trp^{295}$, $Phe^{360}$, $Ser^{361}$, $Pro^{362}$ | (Dong et al., 2012)[23] |
| $Cys^5$ | $Ala^{359}$, $Phe^{360}$, $Glu^{363}$, $Ile^{368}$, $Gln^{369}$, $Phe^{372,}$ $Glu^{373}$ | (Dong et al., 2012)[23] |
| $Cys^6$ | $Ser^{361}$, $Pro^{362}$, $Asp^{364}$, $Ala^{365}$, $Met^{366}$, $Glu^{373}$ | (Dong et al., 2016)[24] |
| $Cys^7$ | $Trp^{295}$, $Phe^{358}$ | (Dong et al, 2016)[24] |

**Supplementary Table 8.** Identification of receptor residues in the three extracellular loops of SecR important for spatial approximation using antagonist Cys-trapping probes.

| Receptor constructs | Cys$^{5}$-(c[E$^{16}$,K$^{20}$],I$^{17}$,Cha$^{22}$,R$^{25}$)sec(5-27) | | Cys$^{6}$-(c[E$^{16}$,K$^{20}$],I$^{17}$,Cha$^{22}$,R$^{25}$)sec(5-27) | | Cys$^{7}$-(c[E$^{16}$,K$^{20}$],I$^{17}$,Cha$^{22}$,R$^{25}$)sec(5-27) | |
|---|---|---|---|---|---|---|
| | Intraloop labeling efficiency (% of max) | Overall labeling efficiency (% of max) | Intraloop labeling efficiency (% of max) | Overall labeling efficiency (% of max) | Intraloop labeling efficiency (% of max) | Overall labeling efficiency (% of max) |
| WT | *N.D.* | *N.D.* | *N.D.* | *N.D.* | *N.D.* | *N.D.* |
| **ECL1** | | | | | | |
| D196C | *N.D.* | < 1 | *N.D.* | < 1 | *N.D.* | < 1 |
| A197C | *N.D.* | < 1 | *N.D.* | < 1 | *N.D.* | < 1 |
| V198C | *N.D.* | < 1 | *N.D.* | < 1 | *N.D.* | < 1 |
| L199C | *N.D.* | < 1 | *N.D.* | < 1 | *N.D.* | < 1 |
| F200C | *N.D.* | < 1 | *N.D.* | < 1 | *N.D.* | < 1 |
| S201C | *N.D.* | < 1 | *N.D.* | < 1 | *N.D.* | < 1 |
| S202C | *N.D.* | < 1 | *N.D.* | < 1 | *N.D.* | < 1 |
| D203C | *N.D.* | < 1 | *N.D.* | < 1 | *N.D.* | < 1 |
| D204C | *N.D.* | < 1 | *N.D.* | < 1 | *N.D.* | < 1 |
| V205C | *N.D.* | < 1 | *N.D.* | < 1 | *N.D.* | < 1 |
| T206C | *N.D.* | < 1 | *N.D.* | < 1 | *N.D.* | < 1 |
| Y207C | *N.D.* | < 1 | *N.D.* | < 1 | *N.D.* | < 1 |
| D209C | *N.D.* | < 1 | *N.D.* | < 1 | *N.D.* | < 1 |
| A210C | *N.D.* | < 1 | *N.D.* | < 1 | *N.D.* | < 1 |
| H211C | *N.D.* | < 1 | *N.D.* | < 1 | *N.D.* | < 1 |
| R212C | *N.D.* | < 1 | *N.D.* | < 1 | *N.D.* | < 1 |
| A213C | *N.D.* | < 1 | *N.D.* | < 1 | *N.D.* | < 1 |
| G214C | *N.D.* | < 1 | *N.D.* | < 1 | *N.D.* | < 1 |
| K216C | *N.D.* | < 1 | *N.D.* | < 1 | *N.D.* | < 1 |
| L217C | *N.D.* | < 1 | *N.D.* | < 1 | *N.D.* | < 1 |
| V218C | *N.D.* | < 1 | *N.D.* | < 1 | *N.D.* | < 1 |
| M219C | *N.D.* | < 1 | *N.D.* | < 1 | *N.D.* | < 1 |
| V220C | *N.D.* | < 1 | *N.D.* | < 1 | *N.D.* | < 1 |
| L221C | *N.D.* | < 1 | *N.D.* | < 1 | *N.D.* | < 1 |
| F222C | *N.D.* | < 1 | *N.D.* | < 1 | *N.D.* | < 1 |
| Q223C | *N.D.* | < 1 | *N.D.* | < 1 | *N.D.* | < 1 |
| **ECL2** | | | | | | |
| F279C | 18.3 ± 3.7 | 5.6 ± 1.7 | 32.1 ± 11.6 | 12.6 ± 5.7 | 17.6 ± 7.2 | 6.6 ± 2.9 |
| L280C | 23.0 ± 3.1 | 7.4 ± 2.3 | 39.4 ± 11.5 | 14.3± 4.6 | 58.0 ± 11.2 | 16.4 ± 4.1 |
| E281C | 11.7 ± 6.6 | 4.4 ± 3.2 | 20.8 ± 7.1 | 7.4 ± 2.8 | 39.8 ± 16.1 | 12.0 ± 5.1 |
| D282C | 9.0 ± 9.0 | 3.9 ± 3.9 | 12.5 ± 4.1 | 4.7 ± 1.5 | 26.0 ± 14.5 | 8.2 ± 4.7 |
| V283C | 15.3 ± 6.5 | 5.5 ± 3.3 | 16.2 ± 5.7 | 5.8 ± 2.4 | 26.3 ± 13.9 | 8.9 ± 5.4 |
| G284C | 12.9 ± 6.6 | 4.8 ± 3.1 | 14.2 ± 5.4 | 4.8 ± 1.6 | 27.8 ± 12.5 | 8.4 ± 5.8 |
| W286C | 14.0 ± 8.7 | 5.5 ± 4.0 | 12.3 ± 4.9 | 4.2 ± 1.3 | 22.3 ± 12.5 | 9.1 ± 5.5 |
| D287C | 18.6 ± 4.8 | 6.3 ± 2.9 | 14.7 ± 5.8 | 5.0 ± 1.9 | 33.7 ± 11.0 | 11.0 ± 5.5 |
| I288C | 22.2 ± 1.7 | 6.9 ± 1.8 | 30.1 ± 5.4 | 11.7 ± 2.4 | 59.3 ± 17.5 | 17.3 ± 8.1 |
| N289C | 44.6 ± 16.4 | 16.4 ± 4.6 | 28.0 ± 4.4 | 5.8 ± 2.0 | 30.3 ± 12.1 | 9.7 ± 5.9 |
| A290C | 52.2 ± 9.8 | 21.6 ± 3.2 | 21.3 ± 9.1 | 6.9 ± 2.5 | 59.5 ± 12.0 | 16.3 ± 4.8 |
| N291C | 59.0 ± 11.0 | 24.5 ± 5.5 | 30.7 ± 14.1 | 9.7 ± 3.9 | 76.2 ± 10.8 | 21.6 ± 6.4 |
| A292C | 78.2 ± 6.4 | 23.4 ± 2.9 | 41.7 ± 11.4 | 14.7 ± 2.1 | 72.3 ± 9.5 | 22.3 ± 6.7 |
| S293C | 89.1 ± 5.5 | 27.0 ± 4.4 | 23.9 ± 9.2 | 8.0 ± 2.3 | 73.4 ± 7.9 | 23.5 ± 5.5 |
| I294C | 84.6 ± 12.7 | 26.8 ± 8.3 | 43.6 ± 14.3 | 14.9 ± 3.0 | 81.2 ± 5.8 | 23.5 ± 5.6 |
| W295C | 76.5 ± 13.5 | 23.8 ± 6.8 | 100.0 ± 0.0 | 40.8 ± 7.0 | 77.9 ± 9.6 | 22.9 ± 5.8 |
| W296C | 45.9 ± 18.7 | 23.9 ± 8.5 | 34.9 ± 2.8 | 13.7 ± 1.5 | 62.6 ± 5.8 | 17.3 ± 3.6 |
| I297C | 42.9 ± 7.4 | 16.9 ± 5.4 | 24.4 ± 7.7 | 8.4 ± 1.9 | 47.5 ± 7.5 | 14.3 ± 4.9 |
| **ECL3** | | | | | | |
| F358C | 74.4 ± 14.5 | 74.4 ± 14.5* | 93.1 ± 2.8 | 93.1 ± 2.8* | 95.0 ± 4.1 | 95.0 ± 4.1* |
| A359C | 87.8 ± 6.3 | 87.8 ± 6.3* | 99.3 ± 0.7 | 99.3 ± 0.7* | 84.2 ± 13.0 | 84.2 ± 13.0* |
| F360C | 63.3 ± 8.1 | 63.3 ± 8.1* | 70.7 ± 4.0 | 70.7 ± 4.0* | 70.5 ± 12.8 | 70.5 ± 12.8* |
| S361C | 87.5 ± 6.3 | 87.5 ± 6.3* | 81.0 ± 3.6 | 81.0 ± 3.6* | 79.8 ± 5.7 | 79.8 ± 5.7* |
| P362C | 30.6 ± 9.7 | 30.6 ± 9.7 | 40.0 ± 2.5 | 40.0 ± 2.5 | 23.8 ± 2.7 | 23.8 ± 2.7 |
| E363C | 22.3 ± 9.1 | 22.3 ± 9.1 | 20.2 ± 1.2 | 20.2 ± 1.2 | 13.7 ± 4.2 | 13.7 ± 4.2 |
| D364C | 22.0 ± 7.7 | 22.0 ± 7.7 | 33.7 ± 2.0 | 33.7 ± 2.0 | 18.7 ± 4.2 | 18.7 ± 4.2 |
| A365C | 22.0 ± 8.1 | 22.0 ± 8.1 | 37.7 ± 3.2 | 37.7 ± 3.2 | 21.5 ± 1.9 | 21.5 ± 1.9 |
| M366C | 17.7 ± 7.9 | 17.7 ± 7.9 | 34.9 ± 1.8 | 34.9 ± 1.8 | 17.5 ± 3.0 | 17.5 ± 3.0 |
| E367C | 4.2 ± 3.0 | 4.2 ± 3.0 | 11.7 ± 4.1 | 11.7 ± 4.1 | 4.9 ± 2.1 | 4.9 ± 2.1 |
| I368C | 13.2 ± 4.9 | 13.2 ± 4.9 | 19.3 ± 5.6 | 19.3 ± 5.6 | 12.2 ± 3.1 | 12.2 ± 3.1 |
| Q369C | 14.2 ± 4.5 | 14.2 ± 4.5 | 41.0 ± 8.0 | 41.0 ± 8.0 | 25.6 ± 6.0 | 25.6 ± 6.0 |
| L370C | 19.2 ± 3.5 | 19.2 ± 3.5 | 43.5 ± 3.4 | 43.5 ± 3.4 | 27.4 ± 4.2 | 27.4 ± 4.2 |
| F371C | 21.8 ± 3.9 | 21.8 ± 3.9 | 39.6 ± 4.9 | 39.6 ± 4.9 | 43.7 ± 9.5 | 43.7 ± 9.5 |
| F372C | 27.3 ± 6.8 | 27.3 ± 6.8 | 46.9 ± 4.7 | 46.9 ± 4.7 | 39.4 ± 6.1 | 39.4 ± 6.1 |
| E373C | 28.2 ± 5.8 | 28.2 ± 5.8 | 62.4 ± 1.4 | 62.4 ± 1.4* | 38.9 ± 4.4 | 38.9 ± 4.4 |
| L374C | 35.0 ± 10.3 | 35.0 ± 10.3 | 88.6 ± 2.8 | 88.6 ± 2.8* | 45.5 ± 12.5 | 45.5 ± 12.5 |

Intraloop labeling efficiency represents intensity of labeling of each band as a percentage of the intensity of the band with the highest labeling intensity within each loop, while overall labeling efficiency represents intensity of labeling of each band as a percentage of the intensity of the band with the highest labeling intensity using that probe in all three loops. Values with underlines and asterisks represent constructs with overall intensities above 25% and 50%, respectively. Values represent means ± SEM of data from a minimum of three independent experiments. *N.D.*, not detectable.

**Supplementary Table 9.** Identification of receptor residues in the juxtamembranous region of the amino-terminal domain important for spatial approximation using antagonist Cys-trapping probes.

| Receptor constructs | $Cys^{5}$-(c[$E^{16}$,$K^{20}$],$I^{17}$,$Cha^{22}$,$R^{25}$)sec(5-27) | | $Cys^{6}$-(c[$E^{16}$,$K^{20}$],$I^{17}$,$Cha^{22}$,$R^{25}$)sec(5-27) | | $Cys^{7}$-(c[$E^{16}$,$K^{20}$],$I^{17}$,$Cha^{22}$,$R^{25}$)sec(5-27) | |
|---|---|---|---|---|---|---|
| | Intrasegment labeling efficiency (% of max) | Overall labeling efficiency (% of max) | Intrasegment labeling efficiency (% of max) | Overall labeling efficiency (% of max) | Intrasegment labeling efficiency (% of max) | Overall labeling efficiency (% of max) |
| WT | *N.D.* | *N.D.* | *N.D.* | *N.D.* | *N.D.* | *N.D.* |
| **TM1** | | | | | | |
| K134C | *N.D.* | < 1 | *N.D.* | < 1 | *N.D.* | < 1 |
| R135C | *N.D.* | < 1 | *N.D.* | < 1 | *N.D.* | < 1 |
| H136C | *N.D.* | < 1 | *N.D.* | < 1 | *N.D.* | < 1 |
| S137C | *N.D.* | < 1 | *N.D.* | < 1 | *N.D.* | < 1 |
| Y138C | *N.D.* | < 1 | *N.D.* | < 1 | *N.D.* | < 1 |
| L139C | *N.D.* | < 1 | *N.D.* | < 1 | *N.D.* | < 1 |
| L140C | *N.D.* | < 1 | *N.D.* | < 1 | *N.D.* | < 1 |
| K141C | *N.D.* | < 1 | *N.D.* | < 1 | *N.D.* | < 1 |
| L142C | *N.D.* | < 1 | *N.D.* | < 1 | *N.D.* | < 1 |
| K143C | *N.D.* | < 1 | *N.D.* | < 1 | *N.D.* | < 1 |
| V144C | *N.D.* | < 1 | *N.D.* | < 1 | *N.D.* | < 1 |
| M145C | *N.D.* | < 1 | *N.D.* | < 1 | *N.D.* | < 1 |
| Y146C | 100 ± 0 | 61 ± 12.2* | *N.D.* | < 1 | 100 ± 0 | 23 ± 5.5 |
| T147C | *N.D.* | < 1 | *N.D.* | < 1 | *N.D.* | < 1 |

Intrasegment labeling efficiency represents intensity of labeling of each band as a percentage of the intensity of the band with the highest labeling intensity within the TM1, while overall labeling efficiency represents intensity of labeling of each band as a percentage of the intensity of the band with the highest labeling intensity using that probe in all three ECLs (see Supplementary Table 8). Value with asterisks represents construct with overall intensity above 50%. Values represent means ± SEM of data from a minimum of three independent experiments. *N.D.*, not detectable.

**Supplementary Table 10.** Main SecR:G protein hydrogen bonds (side chain-side chain) during MD simulations. Data are expressed as the occupancy (% of frames) in which the interactions were present. $^{B}$ indicates residues located at the Gβ subunit.

| G protein residue | Secretin receptor residue | Occupancy (%frames) |
|---|---|---|
| **D381** | $K323^{5.64}$ | 79.2 |
| **$D312^{B}$** | $K401^{8.48}$ | 62.2 |
| **R385** | $E328^{ICL3}$ | 54.7 |
| **R385** | $E328^{ICL3}$ | 54.1 |
| **E392** | $R342^{6.40}$ | 53.5 |
| **$D312^{B}$** | $R169^{ICL1}$ | 44.2 |
| **R342** | $E328^{ICL3}$ | 32.3 |
| **R342** | $E328^{ICL3}$ | 32.2 |
| **Q384** | $K323^{5.64}$ | 30.6 |
| **R38** | $E251^{ICL2}$ | 28.4 |
| **R38** | $E251^{ICL2}$ | 27.6 |

| | | |
|---|---|---|
| **D343** | R330 $^{ICL3}$ | 26.5 |
| **R385** | T326 $^{ICL3}$ | 19.4 |
| **R385** | Q327 $^{ICL3}$ | 13.0 |
| **T350** | E333$^{6.31}$ | 11.8 |
| **R380** | S247$^{ICL2}$ | 11.6 |
| **E322** | R330 $^{ICL3}$ | 11.2 |
| **E392** | R339$^{6.37}$ | 10.9 |
| **Q384** | S247 $^{ICL2}$ | 8.0 |
| **D381** | R322$^{5.63}$ | 7.1 |
| **D323** | R325 $^{ICL3}$ | 6.5 |
| **Y391** | E236$^{3.50}$ | 5.9 |
| **Q35** | E251$^{ICL2}$ | 5.6 |

**Supplementary Table 11.** Main SecR:G protein hydrogen bonds (side chain-backbone) during MD simulations. Data are expressed as the occupancy (% of frames) in which the interactions were present. (bb) indicates residues involved at the backbone atoms level (if both of the two residues are indicated with bb, then alternated side chain-backbone interactions occurred during MD); (Ct) indicates residues involved at the C terminus. $^{B}$indicates residues located in the Gβ subunit. All other interactions are to the Gα subunit.

| G protein Residue | SecR Residue | Occupancy (%frames) |
|---|---|---|
| **L394(Ct)** | R339$^{6.37}$ | 92.9 |
| **Q384** | L244$^{3.58}$(bb) | 53.4 |
| **L393(bb)** | S343$^{6.41}$ | 44.9 |
| **E392(bb)** | R342$^{6.40}$ | 33.5 |
| **R380** | L244$^{3.58}$(bb) | 22.3 |
| **G310$^{B}$(bb)** | Q405$^{8.52}$ | 15.8 |
| **R385** | K323$^{5.64}$(bb) | 15.8 |
| **H387** | L243$^{3.57}$(bb) | 14.3 |
| **T350(bb)** | R330$^{ICL3}$(bb) | 13.8 |
| **K216(bb)** | S250$^{ICL2}$ | 12.2 |
| **R380** | A245$^{3.59}$(bb) | 10.8 |
| **R380** | I246$^{3.60}$(bb) | 8.6 |
| **Q35** | E251$^{4.38}$(bb) | 7.0 |
| **L394(Ct)** | N322$^{6.30}$ | 6.5 |

**Supplementary Table 12.** SecR:G protein generic contacts during MD simulations. Contacts are determined as the occupancy (% of frames) in which at least two atoms were in contact.
$^{B}$ indicates residues located at the Gβ subunit.

| G protein Residue | Secretin receptor Residue | Occupancy (%frames) |
|---|---|---|
| **L394** | R339$^{6.37}$ | 93.2 |
| **L393** | S343$^{6.41}$ | 89.5 |
| **Q384** | L244$^{3.58}$ | 84.5 |
| **H387** | L243$^{3.57}$ | 81.6 |
| **D381** | K323$^{5.64}$ | 79.5 |
| **Y391** | R174$^{2.46}$ | 72.2 |
| **L393** | L320$^{5.61}$ | 66.2 |
| **Y391** | L240$^{3.54}$ | 64.4 |
| **E392** | R342$^{6.40}$ | 63.9 |
| **D312$^{B}$** | K401$^{8.48}$ | 63.2 |
| **R385** | E328 $^{ICL3}$ | 54.4 |
| **R385** | K323$^{5.64}$ | 54.0 |
| **E392** | L346$^{6.44}$ | 53.3 |
| **Q384** | K323$^{5.64}$ | 51.8 |
| **T350** | R330 $^{ICL3}$ | 50.4 |
| **R380** | S247 $^{ICL2}$ | 49.1 |
| **Y391** | L243$^{3.57}$ | 48.7 |
| **D312$^{B}$** | R169 $^{ICL1}$ | 47.7 |
| **H387** | L244$^{3.58}$ | 45.1 |
| **F376** | F248 $^{ICL2}$ | 44.4 |
| **R38** | L244$^{3.58}$ | 40.0 |
| **V217** | F248 $^{ICL2}$ | 39.4 |
| **I383** | S247 $^{ICL2}$ | 39.1 |
| **L393** | L347$^{6.45}$ | 37.1 |
| **H41** | F248 $^{ICL2}$ | 36.1 |
| **R380** | L244$^{3.58}$ | 34.4 |
| **Y391** | Y239$^{3.53}$ | 34.1 |
| **R342** | E328 $^{ICL3}$ | 32.2 |
| **R38** | E251$^{4.38}$ | 32.1 |
| **L393** | L346$^{6.44}$ | 31.8 |
| **R380** | F248 $^{ICL2}$ | 30.8 |

**Supplementary Table 13.** CryoEM collection parameters and refined structure statistics.

| | EMD-21683; PDB 6WI9 | EMD-21972; PDB 6WZG |
|---|---|---|
| **Data Collection** | SecR:sec:DNG$\alpha_s$v1:Nb35 | SecR:sec:DNG$\alpha_s$v2:Nb35 |
| Micrographs | 6500 | 5600 |
| Particles (Final map) | 78k | 350k |
| Pixel size (Å) | 0.86 | 0.83 |
| Defocus range (µm) | 0.5-1.9 | 0.7-1.5 |
| Voltage (kV) | 300 | 300 |
| Electron dose (e/Å$^2$) | 46 | 52.9 |
| Resolution (0.143 FSC) (Å) | 4.3 | 2.3 (tight), 2.4 (wide) |
| **Refinement** | | |
| $CC_{map\_model}$ | 0.78 | 0.71 |
| **Model Quality** | | |
| *RMSD* | | |
| Bond length (Å) / Bond angles (°) | 0.001/0.396 | 0.012/1.586 |
| *Ramachandran* | | |
| Favoured (%) | 95.72 | 98 |
| Outliers (%) | 0 | 0 |
| *Rotamer outliers* (%) | 0 | 1.65 |
| *C-Beta deviations* (%) | 0 | 1.65 |
| *Clashscore* | 5.29 | 4.8 |
| *MolProbity Score* | 1.58 | 1.41 |

**Supplementary Table 14**. List of primers used to prepare secretin receptor constructs with mutations in the TM1 stalk region. Shown are the forward and reverse primer sequences.

| Constructs | Forward primer sequence | Reverse primer sequence |
|---|---|---|
| SecR (K134C) | CTTCCAACGAGTGCCGGCACTCCTAC | GTAGGAGTGCCGGCACTCGTTGGAAG |
| SecR (R135C) | CAACGAGAAGTGTCACTCCTAC | GTAGGAGTGACACTTCTCGTTG |
| SecR (H136C) | CAACGAGAAGCGGTGCTCCTACCTG | CAGGTAGGAGCACCGCTTCTCGTTG |
| SecR (S137C) | GAGAAGCGGCACTGCTACCTGCTGAAG | CTTCAGCAGGTAGCAGTGCCGCTTCTC |
| SecR (Y138C) | CACTCCTGCCTGCTGAAGCTGAAAG | CTTTCAGCTTCAGCAGGCAGGAGTG |
| SecR (L139C) | CTCCTACTGTCTGAAGCTGAAAG | CTTTCAGCTTCAGACAGTAGGAG |
| SecR (L140C) | CTCCTACCTGTGTAAGCTGAAAG | CTTTCAGCTTACACAGGTAGGAG |
| SecR (K141C) | CTACCTGCTGTGTCTGAAAGTC | GACTTTCAGACACAGCAGGTAG |
| SecR (L142C) | CTGCTGAAGTGTAAAGTCATGTAC | GTACATGACTTTACACTTCAGCAG |
| SecR (K143C) | CTGAAGCTGTGTGTCATGTACAC | GTGTACATGACACACAGCTTCAG |
| SecR (V144C) | CTGAAGCTGAAATGCATGTACAC | GTGTACATGCATTTCAGCTTCAG |
| SecR (M145C) | GAAGCTGAAAGTCTGTTACACCGTG | CACGGTGTAACAGACTTTCAGCTTC |
| SecR (Y146C) | CTGAAAGTCATGTGCACCGTGG | CCACGGTGCACATGACTTTCAG |
| SecR (T147C) | GAAAGTCATGTACTGCGTGGGCTACAG | CTGTAGCCCACGCAGTACATGACTTTC |

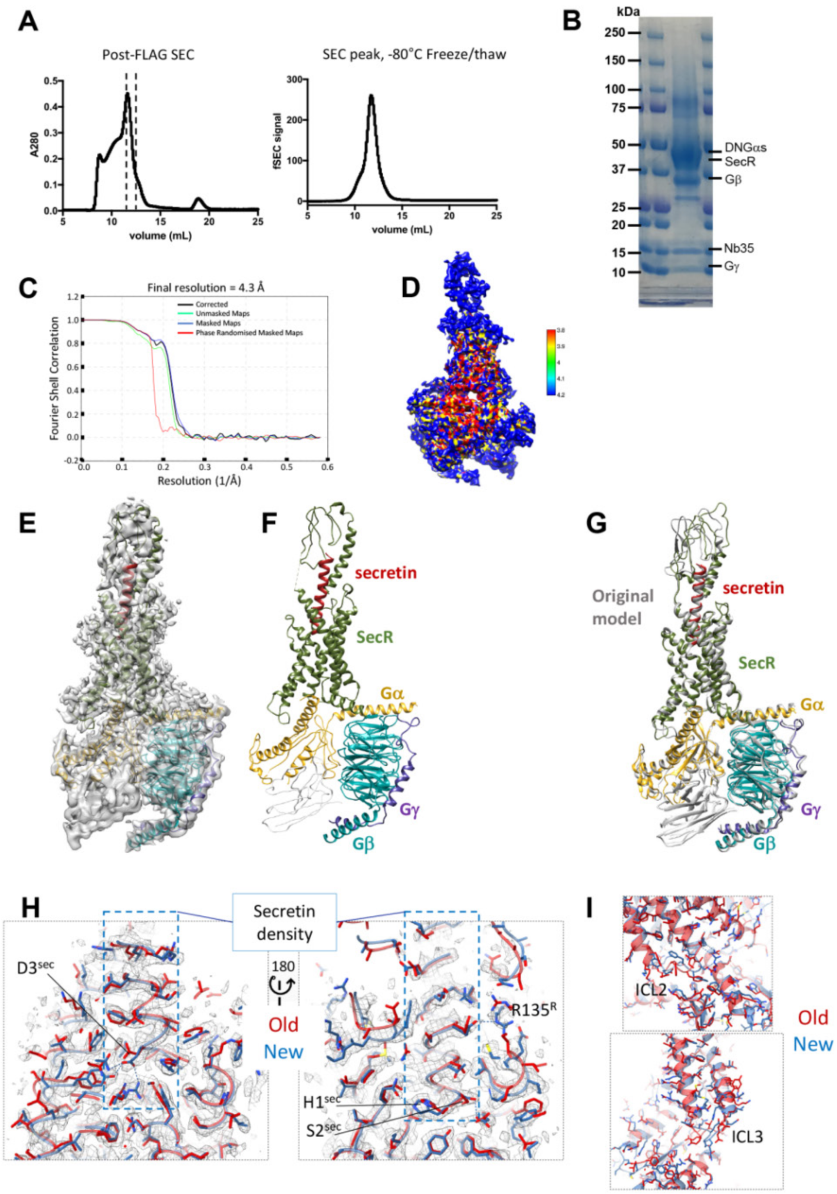


**Supplementary Figure 1.** Initial cryo-EM structure of the secretin:SecR:GsDNv1:Nb35 complex. **A.** SEC trace of post FLAG-affinity column elution (Left panel), the complex peak (dashed lines) was isolated and used for cryo-EM imaging, with the Right panel illustrating stability of the complex following one cycle of freeze-thawing. **B.** Coomassie stain of the purified complex separated by PAGE (Right panel). **C.** Gold standard Fourier shell correlation (FSC) curves for the final map and map validation from half maps, showing the overall nominal resolution of 4.3 Å. **D.** LocalResolution-filtered EM map displaying local resolution (Å) colored from highest resolution (red) to lowest resolution (dark blue). **E.** Full map containing the backbone model of the complex in ribbon format; SecR (olive green), secretin (dark red), G protein α-subunit (gold), β-subunit (cyan), γ-subunit (dark purple), and Nb35 (white). **F.** Ribbon representation of the secretin:SecR:GsDN:Nb35 complex colored according to (**E**). **G**. Overlay of the initial model from the 4.3 Å map (grey) and final pdb model built from the high-resolution maps (Figure 1). **H, I.** Detailed overlay of the initial (red) and final (blue) models together with the high-resolution cryo-EM map density (grey mesh) depicting the secretin peptide-SecR interaction (**H**) and intracellular loops/G protein (**I**), illustrating high concordance between the models.

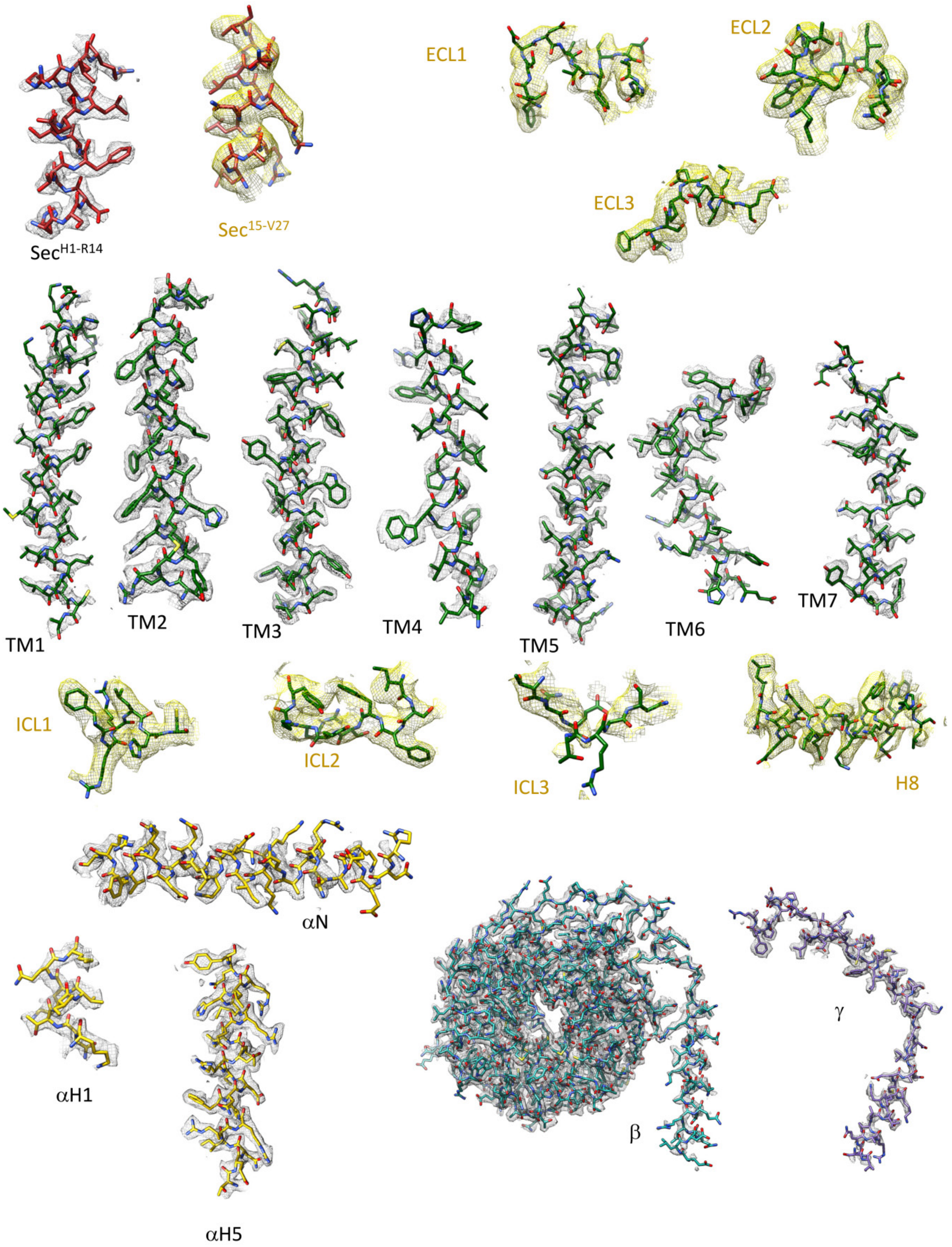


**Supplementary Figure 2.** Atomic modelling of the secretin:SecR:GsDN:Nb35 complex in the cryo-EM density maps. Grey represents the 2.3 Å global map and yellow represents the 2.5 Å receptor-only map (loops). Density maps and models are shown for all seven transmembrane helices, ECL1-3, ICL1-3 and H8 of SecR; the secretin peptide; the N-terminal (αN), αH1, and C-terminal (αH5) α-helices of the Gαs-Ras domain, G$\beta_1$ and G$\gamma_2$ are also shown. The EM map was zoned at 2.0 Å around the protein segments. Images were generated in ChimeraX with a contour setting of 0.032 for the 2.3 Å consensus map, and 0.02 for the 2.5 Å receptor-alone map.

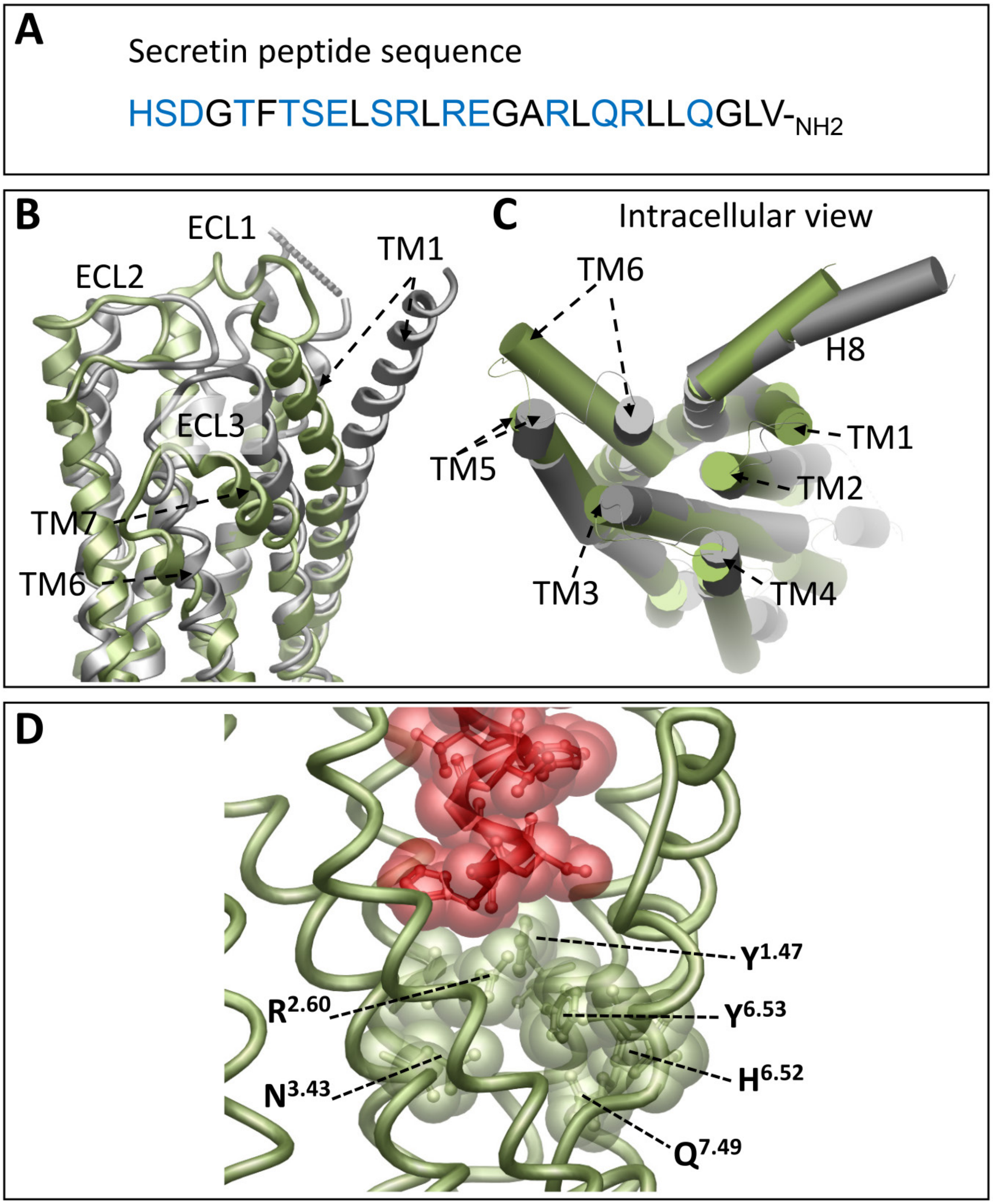


**Supplementary Figure 3. A.** Secretin peptide sequence with polar amino acids highlighted in blue. **B, C.** Comparison of the transmembrane core of the active SecR structure (olive green) and the inactive structure of the related glucagon receptor (dark grey; PDB: 4L6R) highlighting major differences in the location of each of the ECLs and TM1 (**B**, secondary structure displayed in ribbon format) and in the position of the base of TMs 5 and 6 (**C**, helices displayed as cylinders). **D.** The secretin N-terminus is located above the conserved class B central polar network and forms extensive interactions with this network across TM1, TM2, TM5, TM6 and TM7 (see also Supplementary Tables 2-4). Displayed is the consensus cryo-EM derived structural model (PDB 6WZG).

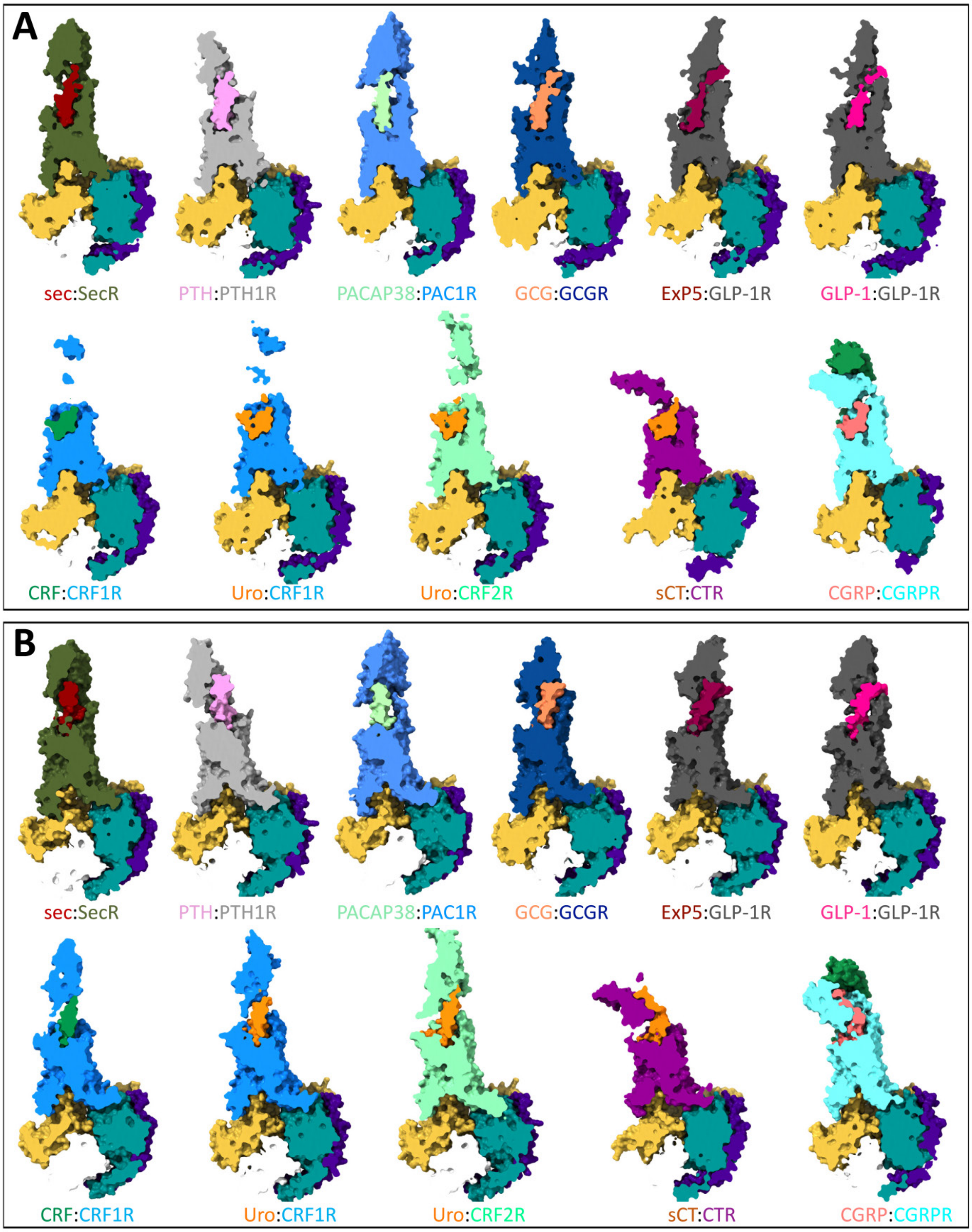


**Supplementary Figure 4.** Comparison of pdb models from cryo-EM maps of available class B active, Gs coupled, receptors. Peptides and receptors are colored according to the legend beneath each figure. G$\alpha$s, gold; G$\beta_1$, dark cyan; G$\gamma_2$, dark slate blue; Nb35, white. **A** and **B** illustrate slices through the models at different levels to highlight either the depth of binding of peptides within the receptor core **(A)** or relative orientation of peptides to ECDs of the receptors **(B)**.

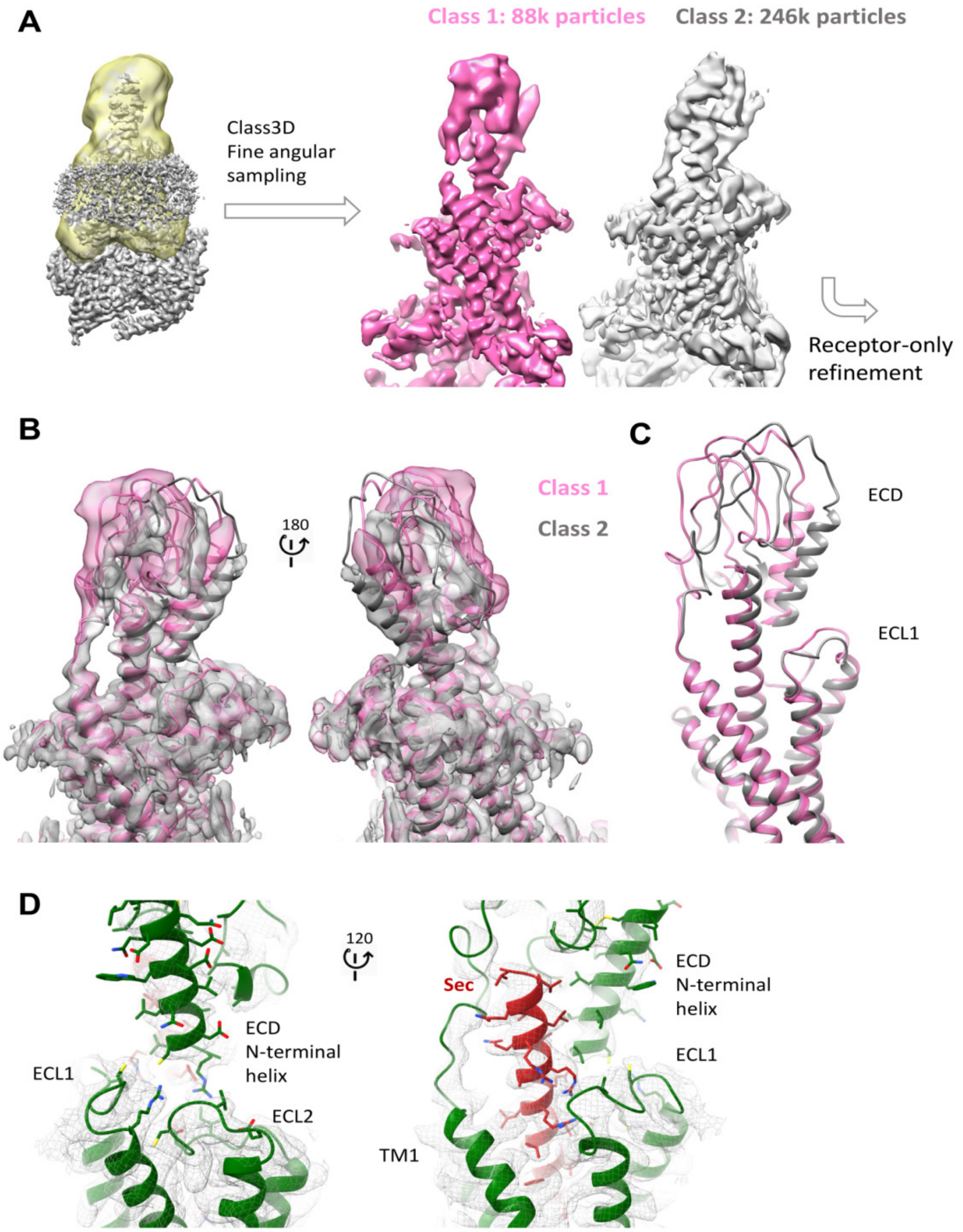


**Supplementary Figure 5.** Variability in receptor-only classification. **(A)** Focused 3D classification using a mask including only the SecR and peptide (yellow) on global refinement particles. 3D classification reveals two predominant classes with variability in the ECD and ECL1 (Class 1 in pink and Class 2 in grey). Particles from Class 2 were further used for the receptor-only refinement. **(B, C)** Backbone models fitted into the two 3D classes (transparent surface representation): the final consensus PDB model is shown in grey. For the Class 1 model, the final PDB model was subjected to a crude flexible fitting of the backbone into the Class 1 map using Namdinator and Isolde (pink). **(D)** Modelling of receptor side chains (x-stick representation) into the SecR electron density map (grey mesh) that are within 6 Å of the far N-terminal helix, illustrating zones of potential interaction. SecR is coloured green, secretin peptide is dark red; the protein backbone is shown in ribbon format. Maps and models are displayed using Chimera (**A-C**) or ChimeraX (**D**).

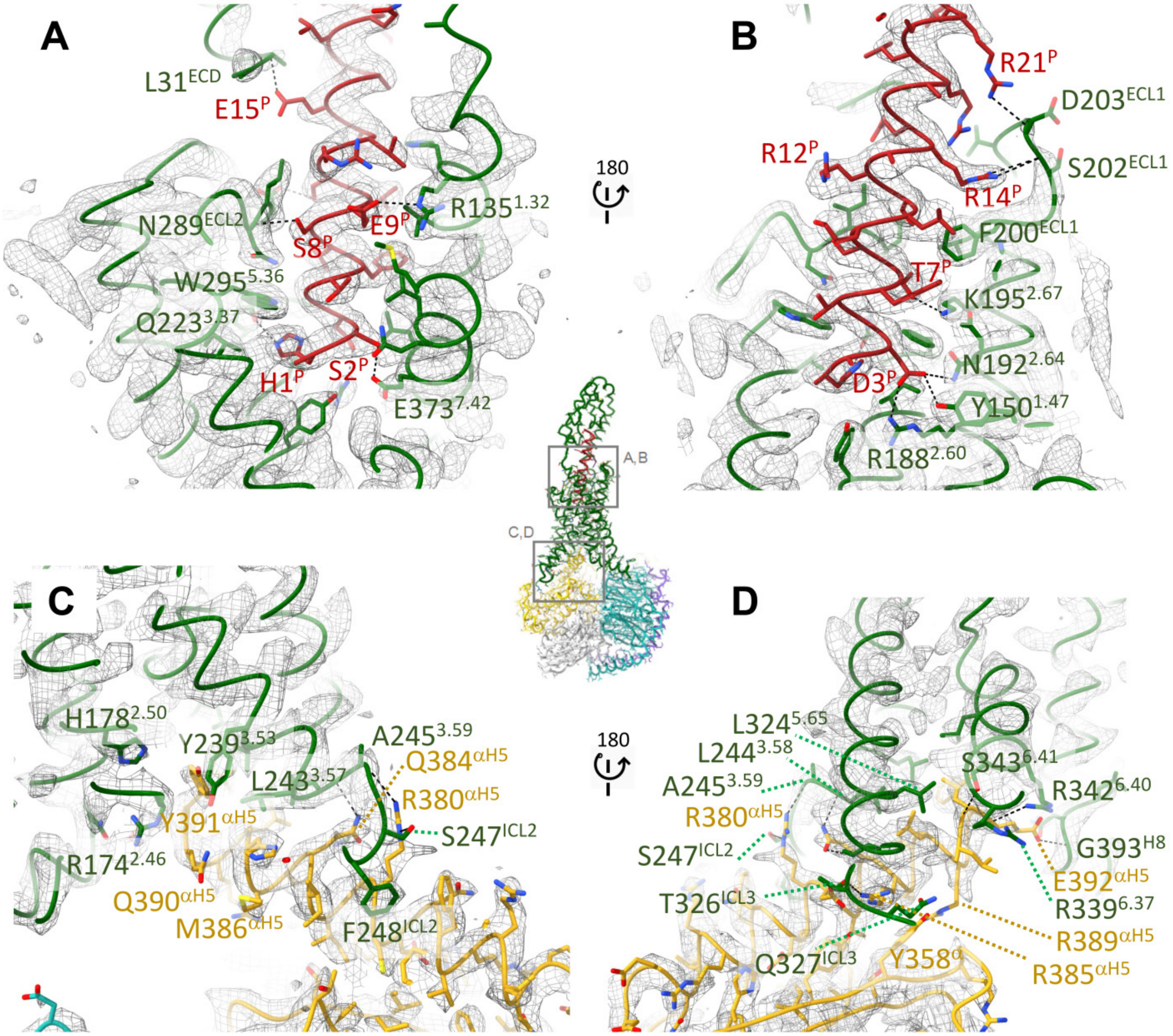


**Supplementary Figure 6.** Interaction of SecR with the secretin peptide and Gα protein. **(A, B)** Cut-away views of the secretin receptor (green) and peptide (red), together with the receptor-only cryo-EM density map (grey mesh). Peptide residues as well as receptor residues within a 4 Å zone around the peptide are shown as stick model. **(C, D):** Cut-away views of SecR (green) and Gα (yellow), together with the cryo-EM density from the global map (grey mesh). Gα C-terminal residues as well as receptor residues within a 4 Å zone around the Gα C-terminus (αH5) are shown as stick model. ChimeraX predicted hydrogen bonds (relaxed distance and angles) between receptor and other chains are shown as black dotted lines.

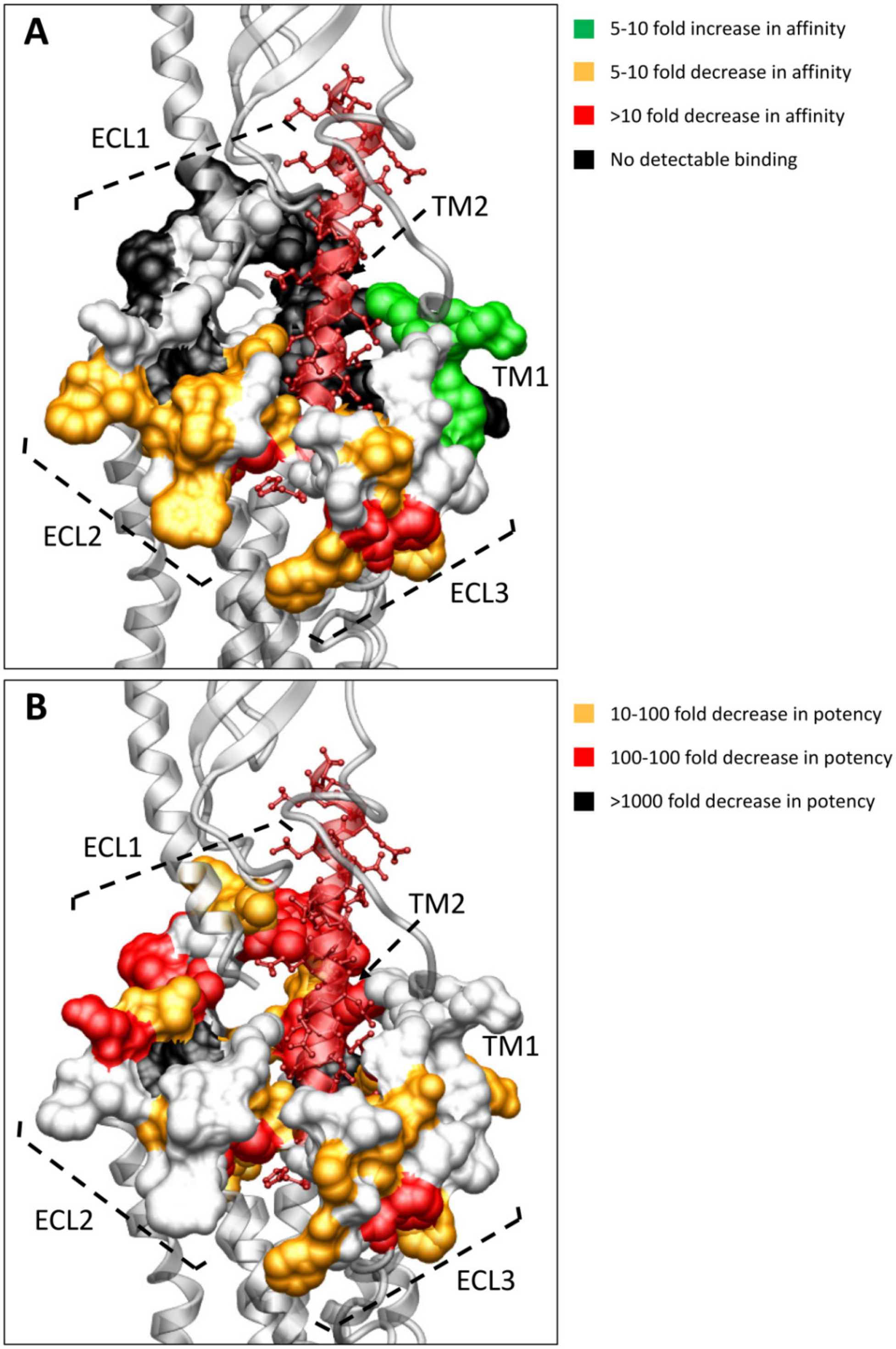


**Supplementary Figure 7.** Effect of cysteine substitution on secretin affinity in competition for $^{125}$I-[Y$^{10}$]secretin(1-27) binding to hSecR **(A)** or secretin-induced cAMP accumulation **(B).** Mutated residues are depicted in grey surface and cpk representation. Colored residues in **(A)** depict 5-10 fold increase (green), 5-10 fold decrease (light orange), >10-fold decrease (red) in affinity, or no detectable binding (black). Colored residues in **(B)** depict >10-fold decrease in potency (light orange), >100-fold decrease in potency (red) or >1000-fold decrease in potency (black). Data are from references[23,24] and Supplementary Tables 5, 6.

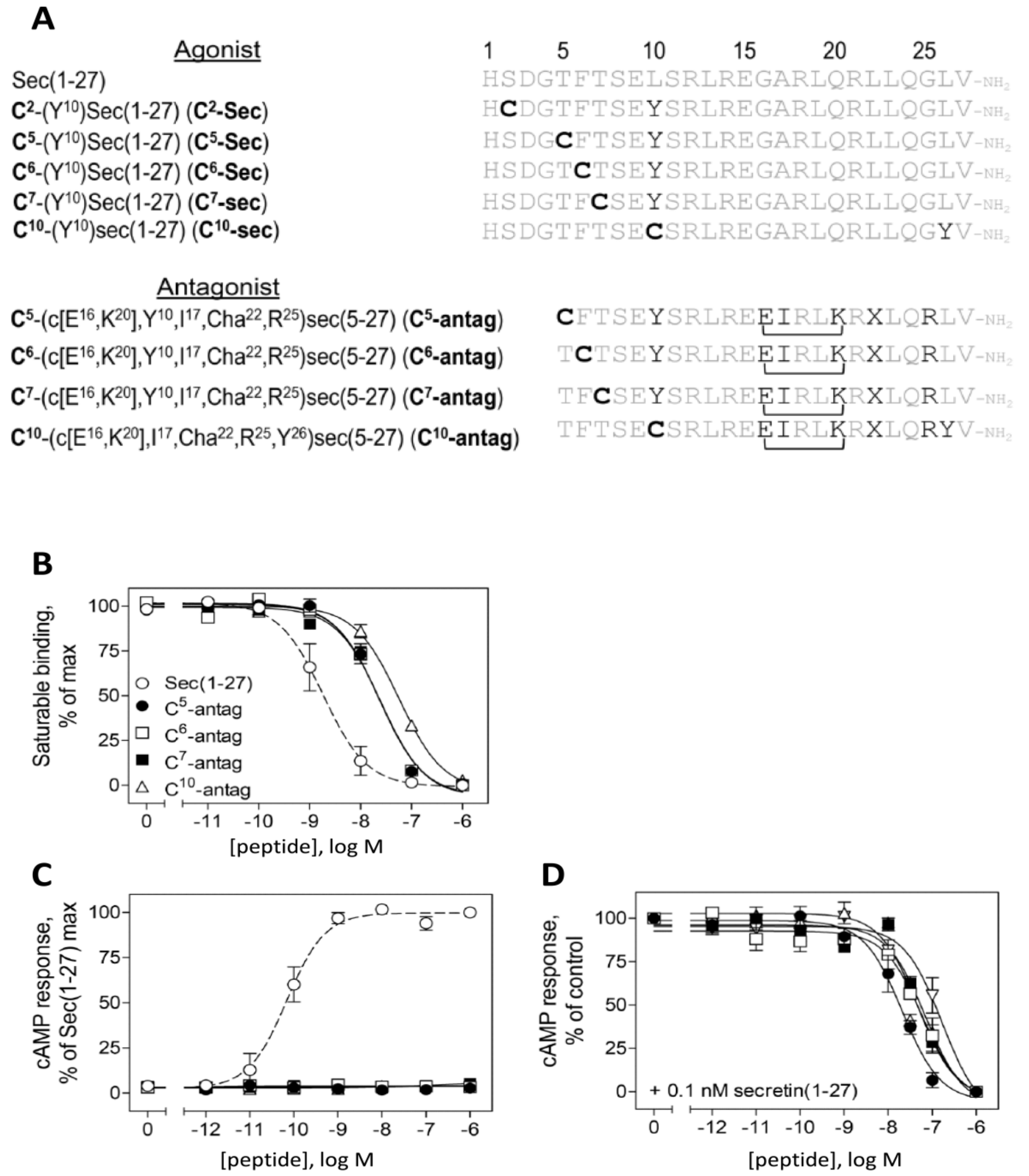


**Supplementary Figure 8. A.** Primary structures of cysteine-containing secretin agonist and antagonist analogues used in this study. Shown are the amino acid sequences of full length secretin(1-27), cysteine containing agonist (Cys$^{2}$-, Cys$^{5}$-, Cys$^{6}$-, Cys$^{7}$- and Cys$^{10}$-sec) that we used previously[31,32] and antagonist (Cys$^{5}$-, Cys$^{6}$-, Cys$^{7}$- and Cys$^{10}$-antag) (developed in this study) probes. Natural residues are colored gray, while modified residues are colored black. Lactam bridges linking the side chains of residues Glu$^{16}$ and Lys$^{20}$ are illustrated with brackets. **B.** Inhibition curves of increasing concentrations of secretin, Cys$^{5}$-, Cys$^{6}$-, Cys$^{7}$- and Cys$^{10}$-antag probes to compete for binding of the secretin radioligand, $^{125}$I-[Tyr$^{10}$]sec(1–27), to secretin receptor-bearing CHO-SecR cells. Values represent the percentages of saturable binding, expressed as the means ± S.E.M. of duplicate values from a minimum of three independent experiments. **C.** Intracellular cAMP accumulation in CHO-SecR cells stimulated with increasing concentrations of each of the antagonist analogues or secretin peptide**.** Data points represent the means ± S.E.M. of data from three independent experiments performed in duplicate. **D.** Inhibition of 0.1 nM secretin mediated cAMP accumulation by increasing concentration of antagonist analogues. Data points represent the means ± S.E.M. of data from three independent experiments performed in duplicate.

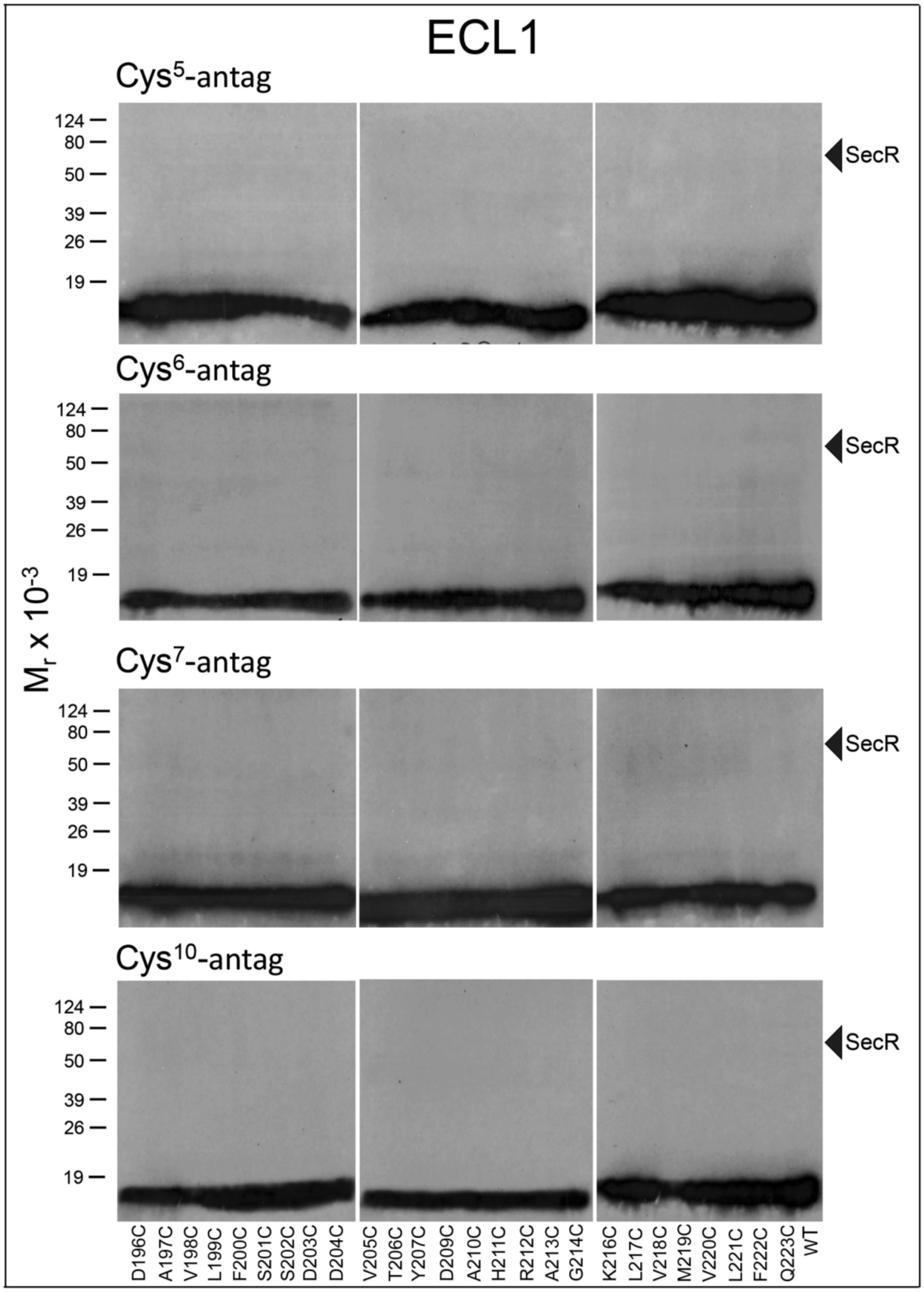


**Supplementary Figure 9.** Cysteine trapping of secretin receptor ECL1 cysteine replacement mutants with $^{125}$I-labelled cysteine-containing secretin antagonist analogues. Shown are typical autoradiographs of 10% SDS-PAGE gels used to separate the products of cysteine trapping of the indicated ECL1 SecR cysteine mutants transiently expressed in COS-1 cells for each of the noted cysteine-containing secretin antagonist probes. Autoradiographs are representative of a minimum of three independent experiments. Since no significant receptor labeling was observed under non-reducing conditions, autoradiographs of reducing gels that also did not show significant labeling are not shown.

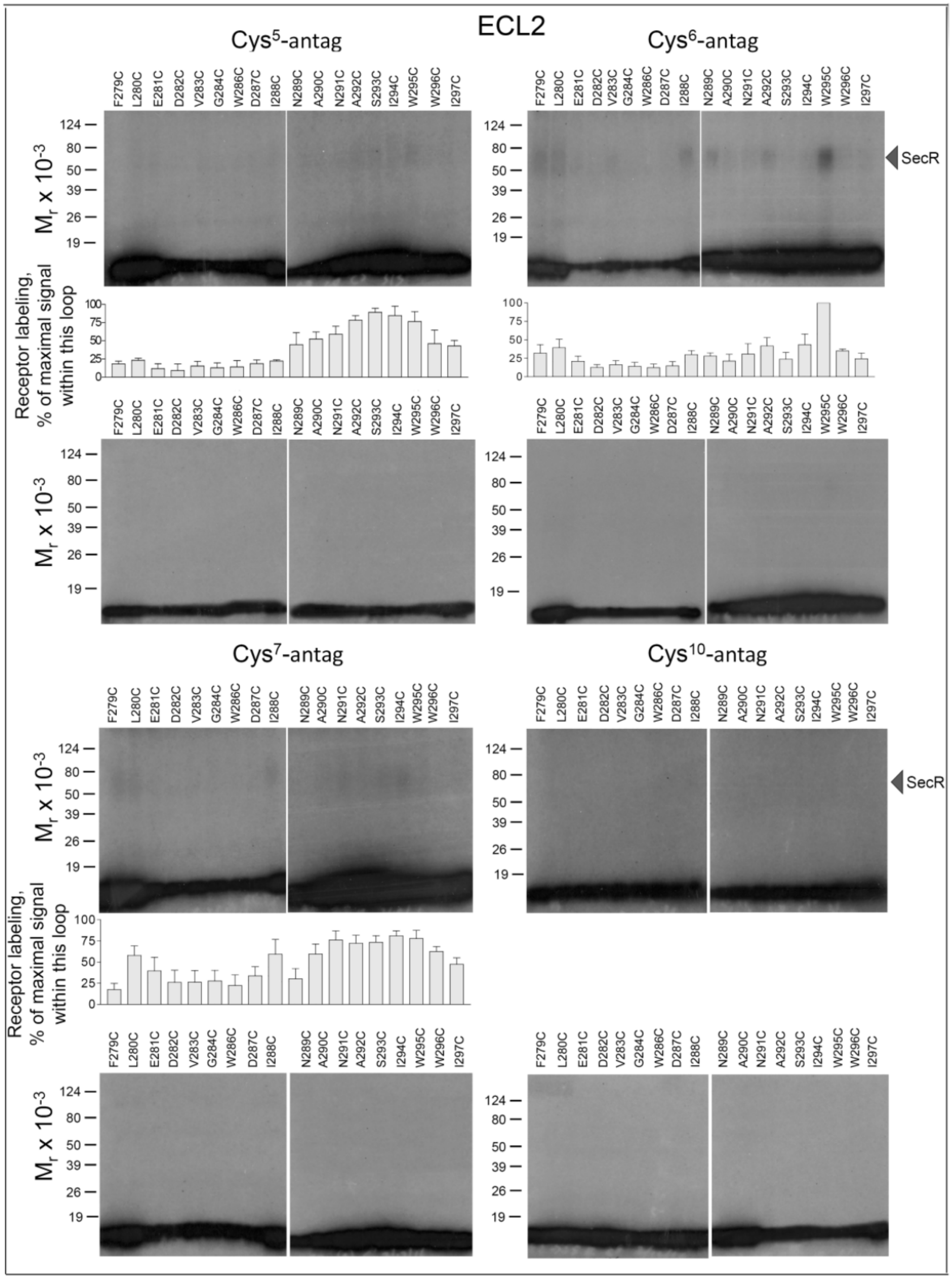


**Supplementary Figure 10.** Cysteine trapping of secretin receptor ECL2 cysteine replacement mutants with $^{125}$I-labelled cysteine-containing secretin antagonist analogues. Shown are typical autoradiographs of 10% SDS-PAGE gels used to separate the products of cysteine trapping of the indicated ECL2 SecR cysteine replacement mutants transiently expressed in COS-1 cells for each of the noted cysteine-containing secretin antagonist probes, under non-reducing (top panel) and reducing (bottom panel) conditions. Autoradiographs are representative of a minimum of three independent experiments. Densitometric analysis of data from three similar experiments with the Cys$^5$-, Cys$^6$-, and Cys$^7$-antagonist probes is shown (middle panel), with intensities representing the percentages of the signal for the maximal labeling of a residue within ECL2 by that probe. Quantification of labeling is not shown for experiments with the Cys$^{10}$-antagonist probe because no significant labeling was observed with any of the receptor cysteine replacement mutants.

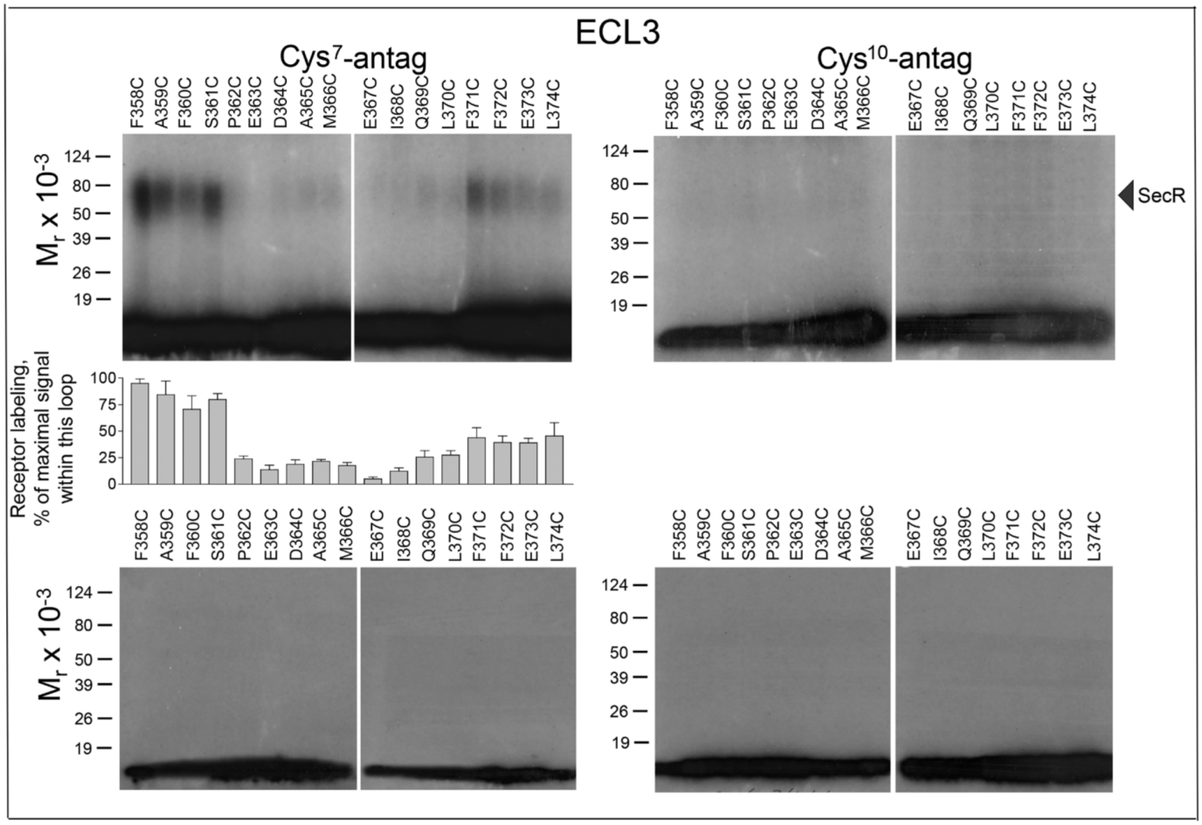


**Supplementary Figure 11.** Cysteine trapping of secretin receptor ECL3 cysteine replacement mutants with $^{125}$I-labelled Cys$^{7}$- or Cys$^{10}$- containing secretin antagonist analogues. Shown are typical autoradiographs of 10% SDS-PAGE gels used to separate the products of cysteine trapping of the indicated ECL3 SecR cysteine replacement mutants transiently expressed in COS-1 cells for each of the noted cysteine-containing secretin antagonist probes, under non-reducing (top panel) and reducing (bottom panel) conditions. Autoradiographs are representative of a minimum of three independent experiments. Densitometric analysis of data from three similar experiments with the Cys$^{7}$-antagonist probe is shown (middle panel), with intensities representing the percentages of the signal for the maximal labeling of a residue within ECL3 by that probe. Quantification of labeling is not shown for experiments with the Cys$^{10}$-antagonist probe because no significant labeling was observed with any of the receptor cysteine replacement mutants.

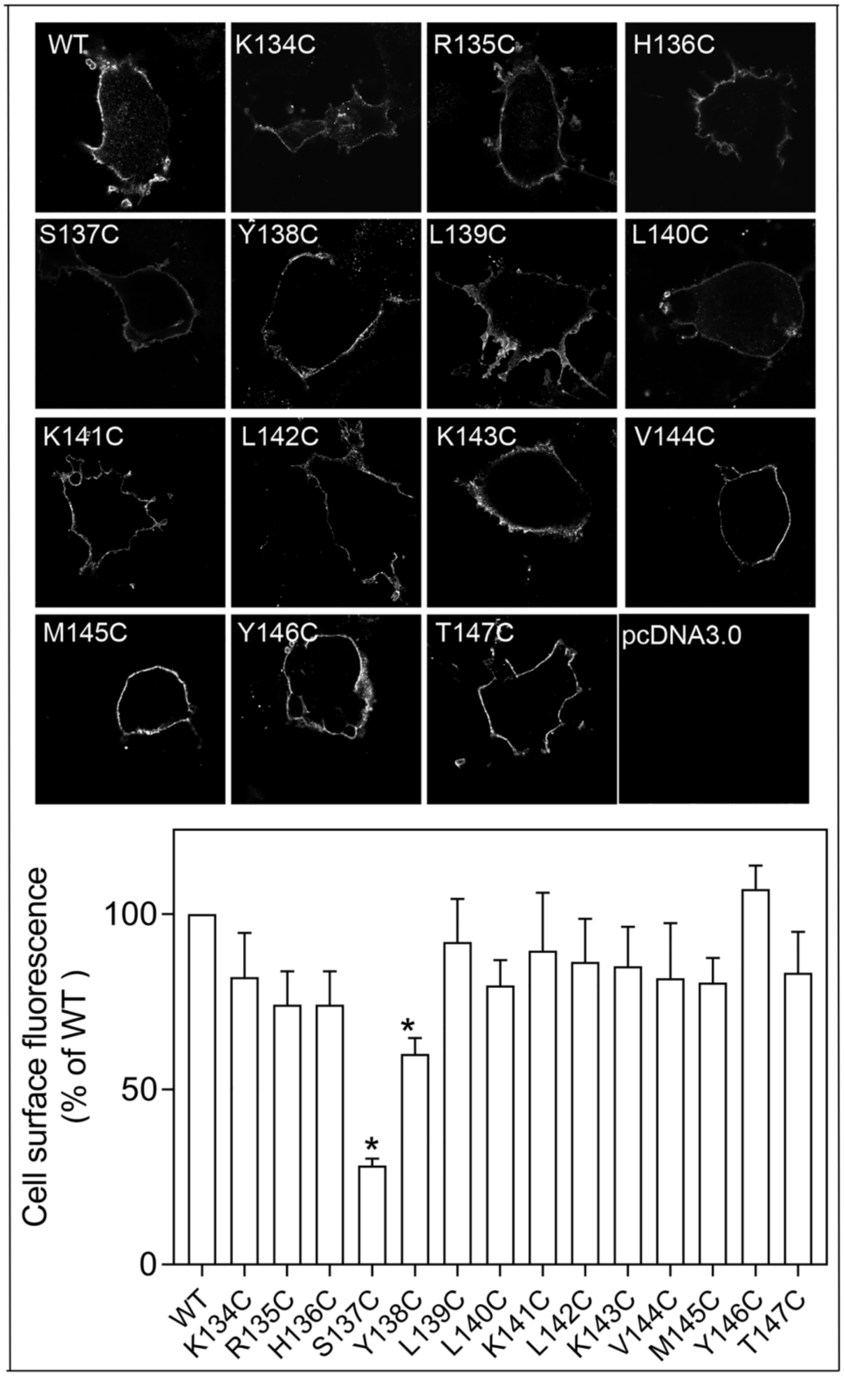


**Supplementary Figure 12.** Morphological evidence of normal surface expression of the cysteine replacement receptor mutants incorporated in each of the positions of the juxtamembranous region of the amino-terminal domain of the secretin receptor. The upper panel shows representative microscopic images of immunostaining of COS-1 cells transiently transfected with wild-type secretin receptor (WT) or noted receptor cysteine replacement constructs, as well as the empty pcDNA3.0 eukaryotic expression vector. Images are representative of three independent experiments. The lower panel shows quantification of receptor cell surface expression as fluorescence percentage of wild type secretin receptor by analyzing 6-8 cells for each of the mutant receptors. The asterisks denote values that are significantly different from that of wild type ($p<0.05$), determined using ANOVA with Dunnett's post-test to analyze the raw data.

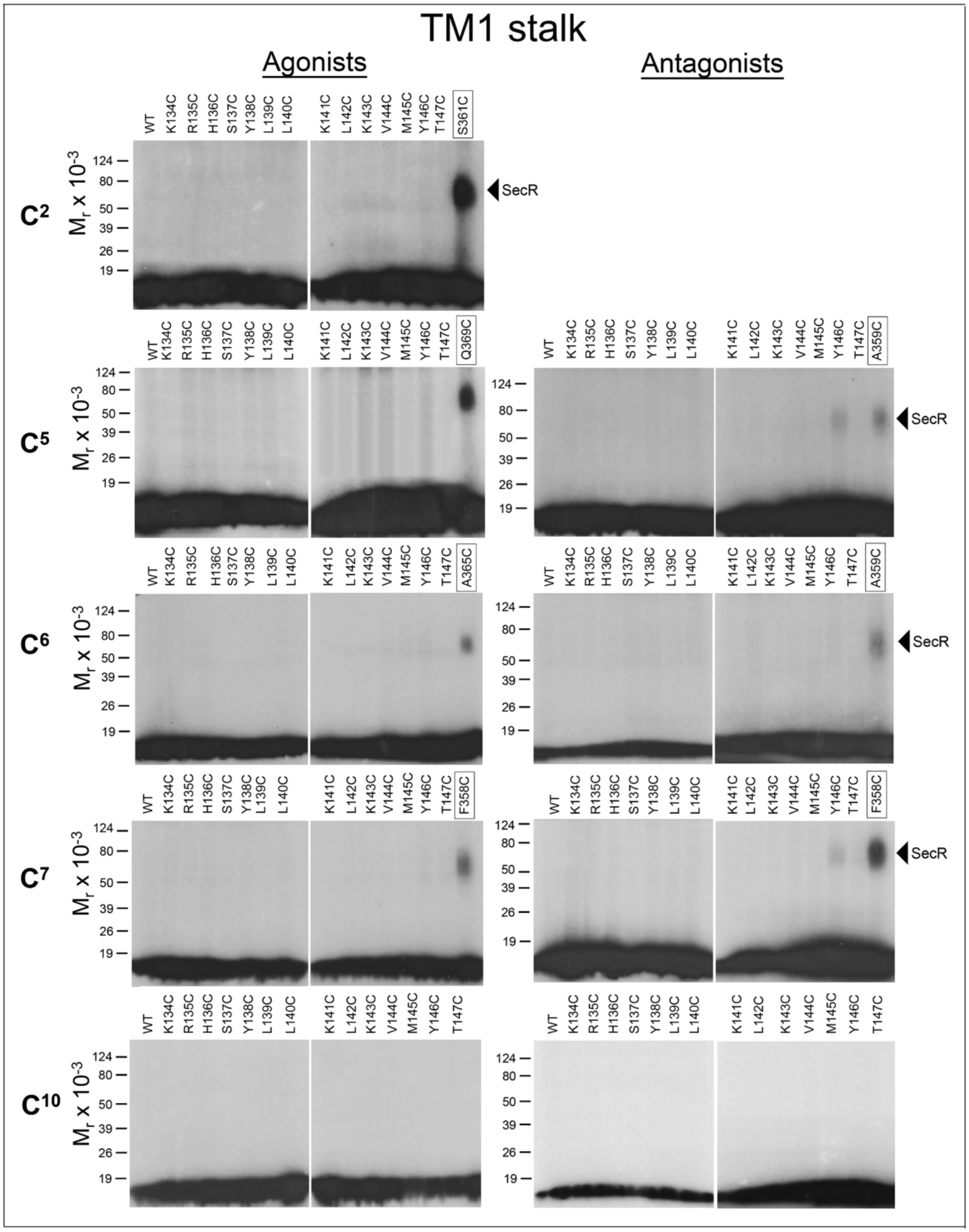


**Supplementary Figure 13.** Cysteine trapping of the cysteine replacement receptor mutants incorporated in each of the positions of the juxtamembranous region of the amino-terminal domain of the secretin receptor with $^{125}$I-labelled cysteine-containing secretin agonist and antagonist analogues. Shown are typical autoradiographs of 10% SDS-PAGE gels used to separate the products of cysteine trapping of indicated SecR cysteine replacement mutants transiently expressed in COS-1 cells with each of the noted cysteine-containing secretin agonist (left panel) and antagonist (right panel) probes. Autoradiographs are representative of a minimum of three independent experiments.

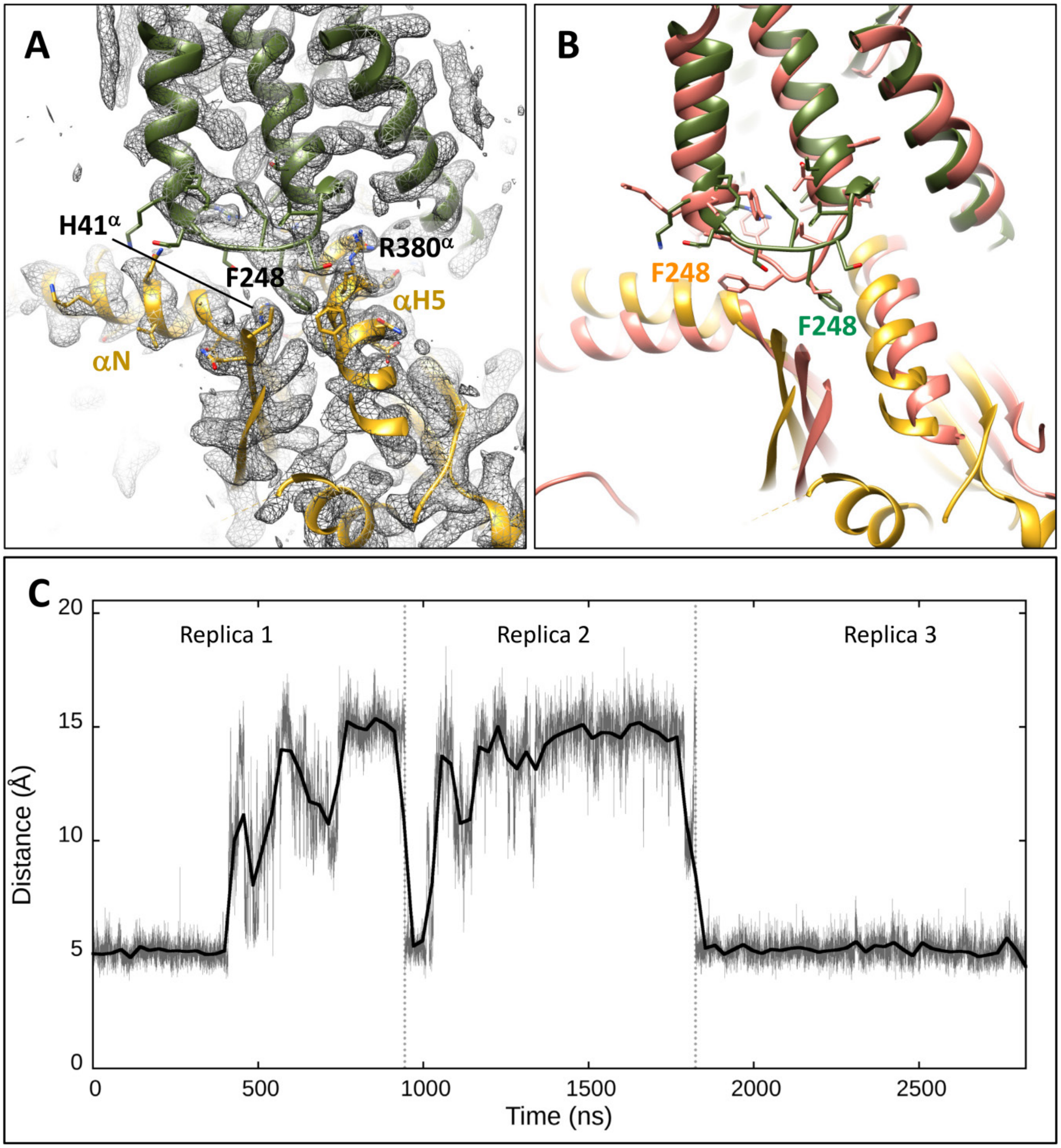


**Supplementary Figure 14.** Conformational variance in the conformation of ICL2 and the G$\alpha$s interface observed in long timescale MD simulations. **A.** EM density and model of the SecR ICL2 – G$\alpha$s interface, illustrating the location of F248$^{ICL2}$ at the junction between the $\alpha$N and $\alpha$5 helices. SecR (green), G$\alpha$s (gold), G$\beta_1$ (cyan) are shown in ribbon format with select residues displayed in x-stick. **B.** Comparison of the ICL2 conformation, and position of F248$^{ICL2}$ relative to G$\alpha$s between the starting conformation from the consensus EM map and at the end of the replica 1 MD simulation (pink colored model) (**Video 4**). **C.** Distance between F248$^{ICL2}$ (SecR) and F387$^{G\alpha}$ benzene rings during MD simulations. Distances close to 5 Å correspond to F248$^{ICL2}$ inserted in the junction between the Gs $\alpha$N helix and $\alpha$5 helix.

.

## Cryosparc 3D Variability pipeline

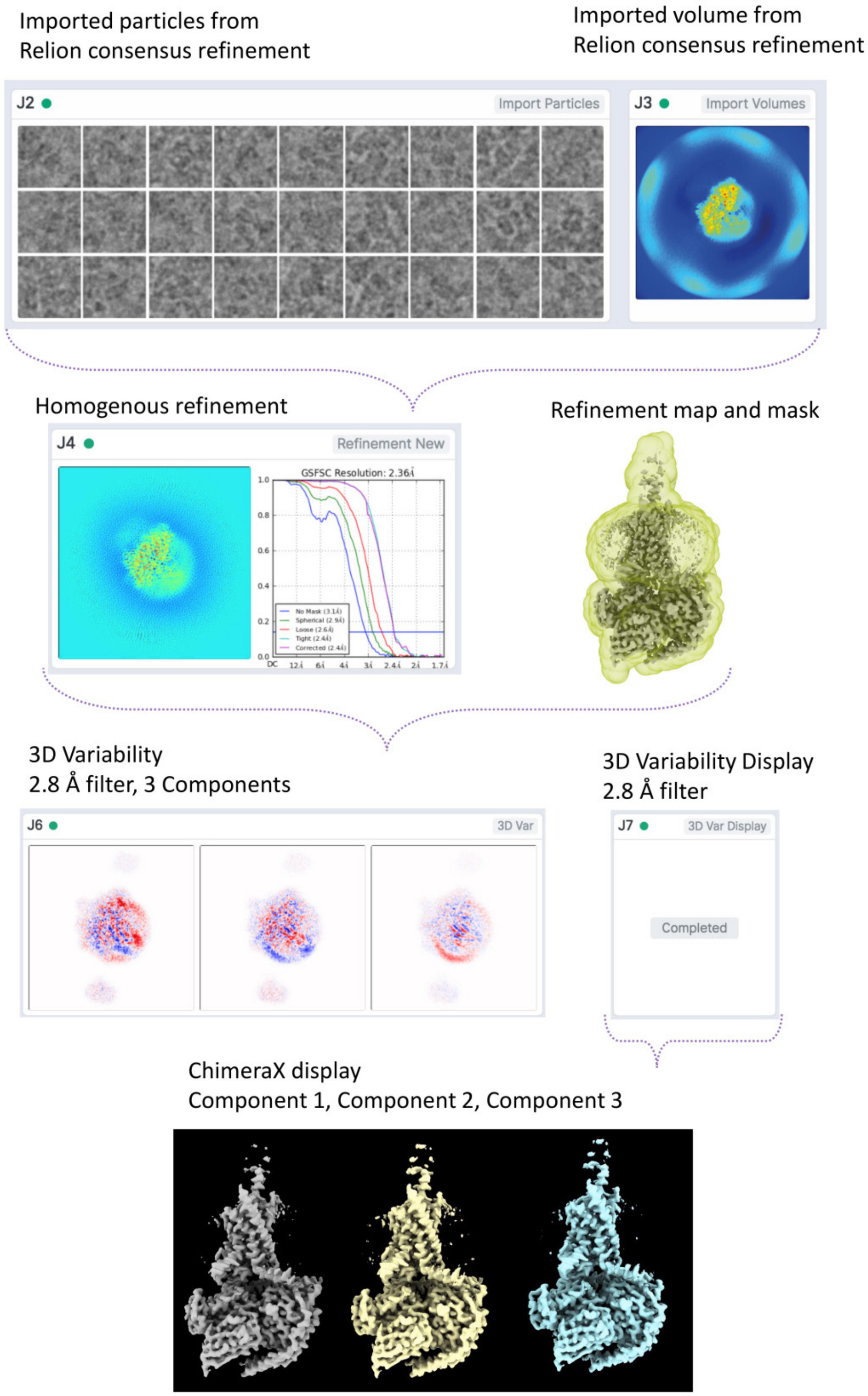


**Supplementary Figure 15.** Cryosparc 3D variability analysis pipeline. Particle stacks and 3D volume from the Relion consensus refinement imported into Cryosparc to generate a homogenous refinement, prior to 3D variability analysis using Cryosparc v2. Outputs were visualized using ChimeraX.
